# Long non‐coding RNA *lncAPAT* promotes atherosclerotic plaque instability by targeting ribosomal protein L22

**DOI:** 10.1002/ctm2.70564

**Published:** 2026-01-13

**Authors:** Rongxia Li, Qiyue Zhang, Yu Chen, Shuting Wang, Shuang Han, Adalaiti Kamili, Yixuan Zhong, Shujun Yang, Weili Zhang

**Affiliations:** ^1^ State Key Laboratory of Cardiovascular Disease, FuWai Hospital, National Center for Cardiovascular Diseases Chinese Academy of Medical Sciences and Peking Union Medical College Beijing China; ^2^ National Clinical Research Center of Cardiovascular Diseases, FuWai Hospital, National Center for Cardiovascular Diseases Chinese Academy of Medical Sciences and Peking Union Medical College Beijing China; ^3^ Department of Cardiovascular Medicine, Xiangya Hospital Central South University Changsha China; ^4^ Center of Coronary Circulation, Xiangya Hospital Central South University Changsha China; ^5^ National Clinical Research Center for Geriatric Disorders, Xiangya Hospital Central South University Changsha China; ^6^ Central China Subcenter of National Center for Cardiovascular Diseases, Henan Cardiovascular Disease Center, Fuwai Central‐China Cardiovascular Hospital Central China Fuwai Hospital of Zhengzhou University Zhengzhou Henan China; ^7^ Institute of Cardiovascular Disease Henan Academy of Innovations in Medical Science Zhengzhou Henan China

**Keywords:** atherosclerosis, cardiovascular disease, inflammation, long non‐coding RNA, macrophage

## Abstract

**Background:**

Long non‐coding RNAs (*lncRNAs*) regulate macrophage inflammation and atherosclerotic plaque stability, but mechanisms need comprehensive investigations.

**Methods:**

Whole‐transcriptome sequencing was used to identify a novel human‐specific lncRNA, *lncAPAT* (atherosclerotic plaque instability‐associated transcript), in the peripheral blood of patients with coronary artery disease (CAD; *n* = 5) with mixed plaques on coronary computed tomography angiography (CCTA). *LncAPAT* was quantified using quantitative real‐time polymerase chain reaction in the discovery cohort and independently validated in patients with coronary mixed plaques by CCTA (*n* = 22) and in patients with acute ST‐segment elevation myocardial infarction (STEMI; *n* = 22). Myeloid cell‐specific *lncAPAT* knock‐in mice were generated and injected with recombinant adeno‐associated virus of murine proprotein convertase subtilisin/kexin type 9 to induce atherosclerosis and explore the effects of *lncAPAT* on inflammation and plaque instability. Macrophages were cultured to evaluate *lncAPAT* function in vitro. Chromatin isolation by RNA purification and sequencing and RNA immunoprecipitation assays were used to identify potential targets of *lncAPAT*.

**Results:**

*LncAPAT* expression was highly expressed in the peripheral blood of CAD and STEMI patients compared with the control individuals. Mice with myeloid cell‐specific *lncAPAT* knock‐in showed an increased plaque burden (2.7‐fold), elevated macrophage counts (2.4‐fold), and higher matrix metalloproteinase (MMP) expression (3.3‐fold for MMP9, 2.0‐fold for MMP2) in thoracic aortic plaques. In vitro, *lncAPAT* significantly promoted the inflammatory responses, adhesive capacity and cholesterol accumulation of macrophages, and decreased the cholesterol efflux ratio. *LncAPAT* interacted with the promoter of the ribosomal protein L22 gene (*RPL22*) and inhibited *RPL22* transcription. *RPL22* inhibition significantly increased the expression of inflammatory cytokines. The RPL22 protein directly interacted with monocyte chemoattractant protein‐1 (*MCP‐1*) mRNA and decreased *MCP‐1* expression. Furthermore, *RPL22* expression in the peripheral blood was lower in CAD and STEMI patients than in control individuals.

**Conclusions:**

*LncAPAT* promoted the macrophage inflammatory response by inhibiting *RPL22* transcriptional activity, contributing to plaque instability.

**Key points:**

1. A novel human‐specific long non‐coding RNA (lncRNA), named as *lncAPAT*, is highly expressed in the peripheral blood of patients with coronary mixed plaques.
2. *LncAPAT* increases thoracic aortic plaque instability and promotes macrophage inflammatory responses.
3. *LncAPAT* directly interacted with the promoter region of *RPL22* to inhibit *RPL22* transcriptional activity and increase the expression of *MCP‐1*, thereby contributing to plaque instability.

## Graphical abstract

*LncAPAT* interacted with the promoter region of *RPL22* to inhibit *RPL22* transcriptional activity and increase the expression of *MCP‐1*, thereby contributing to plaque instability.

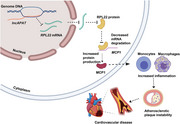

## 1 INTRODUCTION

Atherosclerosis is a chronic inflammatory disorder that underlies cardiovascular disease pathogenesis. Atherosclerotic plaque rupture and thrombosis are clinical manifestations of cardiovascular diseases, including stroke and myocardial infarction. In atherosclerosis, circulating monocytes are recruited to the injured endothelium where they encounter macrophage colony‐stimulating factor, which leads to their differentiation into macrophages and subsequent lipoprotein engulfment.^1^ Macrophages internalise native and modified low‐density lipoprotein through micropinocytosis or scavenger receptor pathways, leading to foam cell formation, a characteristic of atherosclerotic plaques. Foam cells release proinflammatory cytokines and chemokines that exacerbate the inflammatory response, playing a critical role in early atherogenesis and plaque instability.^1^, ^2^ Recent studies have highlighted key epigenetic regulators involved in atherosclerosis development, but the molecular mechanisms that regulate plaque instability have not been thoroughly determined.^3^

Long non‐coding RNAs (*lncRNAs*) are transcripts that exceed 200 nucleotides in length, with minimal protein‐coding capacity. Recent studies have highlighted the clinical relevance of *lncRNAs* in cardiovascular diseases, particularly in terms of their ability to modulate atherosclerotic plaque stability, which is a key determinant of acute cardiovascular events, such as stroke and myocardial infarction. Several *lncRNAs* have been implicated in plaque destabilisation. For example, *PSMB8‐AS1* has been shown to exacerbate atherosclerosis by upregulating *PSMB9* expression, contributing to the inflammatory response and plaque rupture.^4^ Knockdown of *lncRNA RAPIA*, which was highly expressed in advanced atherosclerotic lesions and macrophages, exerted atheroprotective effects through inducing the apoptosis of macrophages.^5^ Moreover, *INKILN* has been reported to enhance vascular smooth muscle cell activation, which is closely linked to fibrous cap thinning and plaque vulnerability.^6^ The roles of specific *lncRNAs* in plaque instability are well appreciated, but further identification and characterisation of novel *lncRNAs* may yield new perspectives about the mechanisms of plaque destabilisation, facilitating the identification of potential therapeutic targets to prevent clinical events.

Coronary artery atherosclerosis (CAD) is considered systemic arteriosclerosis. The resulting coronary artery stenosis can impair blood flow to the heart, causing symptoms such as chest tightness and pain because of luminal narrowing. Coronary computed tomography angiography (CCTA) permits non‐invasive visualisation of the coronary arteries, providing an overall assessment of the atherosclerosis burden and determining plaque volume subtypes within the coronary artery lumen.^7^, ^8^ CCTA has become the primary method used to screen and diagnose coronary artery atherosclerosis because of the abundance of strong outcome and cost‐effectiveness data.^9^ Another imaging option is optical coherence tomography, an invasive high‐resolution imaging technique that effectively delineates plaque characteristics, such as fibrous cap thickness, lipid necrotic core size, and calcification, and it has shown a strong correlation with histological analysis.^10^, ^11^

Herein, we report a novel human‐specific *lncRNA*, *lncAPAT* (atherosclerotic plaque instability‐associated transcript), which was highly expressed in the peripheral blood of CAD patients with coronary mixed plaques according to whole‐transcriptome sequencing. We  explored the effects of *lncAPAT* on inflammation and plaque instability in myeloid cell‐specific *lncAPAT* knock‐in mice and evaluated its function. Moreover, we identified the potential targets of *lncAPAT* and further explored the mechanisms underpinning the involvement of *lncAPAT* in atherosclerosis progression.

## 2 METHODS

The full methodological details can be found in the Supporting Information.

### 2.1 Studied patients

Two independent clinical cohorts were enrolled to investigate *lncAPAT* expression. The participants were not receiving any lipid‐lowering therapy within 3 months before admission. The discovery cohort was designed to identify differentially expressed *lncRNAs* by whole‐transcriptome sequencing, and the findings were confirmed by quantitative real‐time polymerase chain reaction (qRT‐PCR). The discovery cohort comprised 15 subjects: five CAD patients with mixed plaques and 10 control individuals. Subjects who were suspected of having CAD and who were scheduled to undergo CCTA at FuWai Hospital were consecutively enrolled between March and April 2017. Patients with haemopathies, peptic ulcers, hepatic and/or renal insufficiency, infections, autoimmune disease, and/or tumours were excluded. Coronary artery images were acquired using a 64‐detector row CT system (GE Healthcare), which were interpreted by two independent radiologists blinded to the patients’ information. According to the coronary plaque characteristics, the patients were divided into two groups: the control group and the mixed plaque group. Plaques were classified as described previously.^8^ Specifically, plaques ≥1 mm^2^ in structure and distinctly separate from the coronary artery lumen were classified as atherosclerotic plaques (Figure S1), whereas lesions <1 mm^2^ in structure were assigned to the control group. Plaques containing lipid, fibrous, and calcified components were regarded to be mixed plaques. Human peripheral blood samples were collected in Tempus^™^ Blood RNA Tubes after a 12‐h overnight fast and stored at −80°C. The clinical characteristics of participants are presented in Table S1.

A validation cohort was established to confirm *lncAPAT* expression across the plaque subtypes. The validation cohort comprised 81 participants (22 CAD patients with mixed plaques, 22 ST‐segment elevation myocardial infarction [STEMI] patients, and 37 control individuals) enrolled from March 2017 to March 2019 at FuWai Hospital. The control individuals and patients with CAD were recruited using the same criteria as in the discovery cohort. Patients with acute STEMI underwent primary percutaneous coronary intervention and optical coherence tomography for culprit lesions. Patients with shock of cardiac origin, severe hepatic and/or renal insufficiency (estimated glomerular filtration rate <30 mL/min/1.73 m^2^), congestive heart failure (left ventricular ejection fraction <50%), allergy to contrast media, or contraindications to aspirin or ticagrelor were excluded. Peripheral blood samples were collected from STEMI patients upon admission to the emergency department and before the initiation of percutaneous coronary intervention, ensuring that all specimens were obtained prior to reperfusion therapy. Peripheral blood samples were collected in Tempus Blood RNA Tubes and stored at −80°C for qRT‐PCR analysis of *lncAPAT* expression. The clinical characteristics of the participants are shown in Table S2.

To investigate the expression of *lncAPAT* in smooth muscle cells, endothelial cells, and macrophages within atherosclerotic plaques, five human carotid atherosclerotic plaques were obtained from patients undergoing carotid endarterectomy at Fuwai Hospital between March and April 2017. Immediately after excision, the plaques were snap‐frozen in liquid nitrogen and stored at −80°C for subsequent analyses.

This study was conducted in compliance with the Declaration of Helsinki and approved by the Ethics Committee of Fuwai Hospital, with written informed consent obtained from all participants.

### 2.2 Whole‐transcriptome sequencing and data analysis

Total RNA was extracted from peripheral blood using the Tempus^™^ Spin RNA Isolation Kit (Thermo Fisher). Strand‐specific libraries were prepared using the TruSeq^®^ Stranded Total RNA Sample Preparation Kit (Illumina) according to the manufacturer's instructions, followed by sequencing on the Illumina HiSeq X‐ten platform. Differentially expressed *lncRNAs* were identified using the following criteria: false discovery rate ≤.01, fold change ≥2, and fragments per kilobase million (FPKM) ≥1. The protocol details are presented in the Supporting Information.

### 2.3 Northern blot

Northern blot analysis was performed using a DIG Northern Starter Kit (Roche) to confirm the presence of the *lncAPAT* transcript. The protocol details are presented in the Supporting Information.

### 2.4 Fluorescence in situ hybridisation

The Ribo Fluorescent In Situ Hybridization Kit (RiboBio) was used to perform RNA fluorescence in situ hybridisation to determine subcellular localisation of *lncAPAT* in macrophages. The cytoplasmic and nuclear positive controls were *h‐18S* and *h‐U6*, respectively. Images were obtained using a confocal laser scanning microscope (Leica).

### 2.5 Cytoplasmic and nuclear RNA purification

RNA from the cytoplasm and nucleus was separated using a Cytoplasmic & Nuclear RNA Purification Kit (Norgen). The subcellular localisation of *lncAPAT* in macrophages was analysed by the qRT‐PCR method. The localisation of *lncAPAT* in the cytoplasm and nucleus was normalised to ribosomal protein *S14* and *U2* small nuclear ribonucleoprotein, respectively. The primers are listed in Table S3.

### 2.6 Chromatin isolation by RNA purification and sequencing

The chromatin isolation by RNA purification (ChIRP) procedure was carried out using the EZ‐Magna ChIRP RNA Interactome Kit (Millipore). Antisense biotinylated DNA tiling probes corresponding to full‐length *lncAPAT* were designed (RiboBio, China). RNA from the enriched product was isolated with Qiagen miRNeasy^®^ Mini Kit and analysed using iTaq Universal One‐Step Kit (Bio‐Rad). DNA from the enriched product was analysed using the NovaSeq 6000 platform (Illumina). The protocol details are presented in the Supporting Information.

### 2.7 Mouse studies

Myeloid cell‐specific *lncAPAT* knock‐in mice were generated by Cyagen Biosciences. Briefly, the CAG‐loxP‐Stop‐loxP‐APAT‐polyA cassette was cloned into the *Gt (ROSA)26Sor* locus in C57BL/6N mice by CRISPR/Cas9‐mediated gene‐editing technology to generate *lncAPAT* heterozygous mice, which were identified by positive PCR screening. Heterozygous male and female mice were used as founders to generate control mice [C57BL/6N‐Gt (ROSA)26Sor^em1^(APAT)^Cyagen^, *lncAPAT^flox/flox^*]. *LncAPAT* was not expressed in *lncAPAT^flox/flox^* mice because of the ‘stop’ sequence in front of the loxP sites. *LncAPAT^flox/flox^* mice were crossed with *LyzM*‐Cre mice, a strain with myeloid cell‐specific cyclisation recombinase expression, to obtain the experimental mice (myeloid cell‐specific *lncAPAT* expression, *lncAPAT^flox/flox^;LyzM*‐Cre^+/−^). Control and experimental mice were littermates. *LncAPAT^flox/flox^;LyzM*‐Cre^+/−^ male mice were crossed with *lncAPAT^flox/flox^* female mice, yielding approximately equal numbers of control (*lncAPAT^flox/flox^*) and experimental (*lncAPAT^flox/flo^* ^x^ *;LyzM*‐Cre^+/−^) mice. The genotyping methods are presented in the Supporting Information.

The atherosclerosis mouse model in this study was established as Bjorklund's approach.^12^ To induce atherosclerosis, 8‐week‐old male mice were injected with 5.0 × 10^11^ vector genomes recombinant adeno‐associated virus of murine proprotein convertase subtilisin/kexin type 9 (rAAV‐mPCSK9; Vigenebio) via the tail vein, followed by a 12‐week western diet (Research Diets, 1.25% cholesterol and  .5% sodium cholate). At 19 weeks, blood samples were taken from the retro‐orbital sinus, and the serum was separated to measure the concentrations of lipids, mPCSK9, glucose, and transaminase. At the end of the study (at 20 weeks), the mice were euthanised by intraperitoneal injection of pentobarbital (50 mg/kg; Sigma), and the aortae were collected for histological analysis. An atherosclerotic plaque area of approximately 5%–25% in the whole aorta and 20%–70% in the aortic arch was induced at 20 weeks.

Peripheral blood mononuclear cells (PBMCs), bone marrow‐derived macrophages (BMDMs), peritoneal macrophages, and bone marrow neutrophils were isolated and cultured to analyse gene expression. Male mice were primarily used in this study to minimise potential variability arising from sex differences. Nevertheless, to ensure the robustness and generalisability of our findings, the pro‐atherogenic effect of *lncAPAT* and the expression changes of its downstream targets in plaque tissues were independently validated in female mice. A random number table was used for randomisation. The mouse experiments were blinded through numerical coding of the samples. All animal studies were conducted in compliance with FuWai Hospital's Institutional Animal Care and Use Committee and followed Directive 2010/63/EU of the European Parliament for the ethical use of animals in research.

### 2.8 Atherosclerotic lesion analysis

Atherosclerotic lesions in the mouse aorta were assessed by en face analysis and cross‐sectional measurement. Aortic en face lesions were assessed using Oil red O staining (Sigma‐Aldrich). Images were obtained using a stereomicroscope‐dedicated camera (Zeiss). Atherosclerotic lesions in the aorta were expressed as a percentage of the overall surface area.

For the analysis of plaque morphology, fresh isolated heart and aortic tissues were frozen, and 8‐µm‐thick sections (spaced  .5‐mm apart) were continuously cut. The aortic root was identified by the appearance of aortic valve leaflets, and the plaque lesion area was analysed in three consecutive sections for each mouse. Thoracic aortic sections were collected from the most stenotic part of the plaque, and three consecutive sections for each mouse were analysed. Haematoxylin and eosin staining was performed, and lesion size was quantified by a technician blinded to the experimental groupings by morphometric analysis using Image‐Pro Plus software (Media Cybernetics).^13^ Lesion area was recorded as an average of three consecutive sections per mouse.

Plaque composition and lesion stability were also assessed. Oil red O staining was used to assess the lipid content of the plaques, and Picrosirius Red staining was used to assess the ratio of type III/I collagen. Moreover, Masson's trichrome staining was used to assess the total collagen content, and Verhoeff–Van Gieson staining was used to assess elastin. Immunohistochemical staining was used to assess the expression of matrix metalloproteinases (MMPs), α‐smooth muscle actin, and ribosomal protein L22 (RPL22) in macrophages from plaque lesions. The protocol details are presented in the Supporting Information.

### 2.9 Lipid profiling

Serum total cholesterol, triglycerides, transaminase, and glucose were measured in mice using an automatic dry biochemical analyser (Fujifilm). Serum high‐density and low‐density lipoprotein cholesterol concentrations were assessed by a double reagent direct method (Nanjingjiancheng). The protocol details are presented in the Supporting Information.

### 2.10 Cell adhesion assay

Cell adhesion assays were performed to assess the effect of *lncAPAT* on monocyte adhesion to endothelial cell monolayers. The protocol details are presented in the Supporting Information.

### 2.11 Cholesterol accumulation and efflux assays

Oil red O staining was used to evaluate macrophage cholesterol accumulation, and the cholesterol efflux assay was used to assess macrophage cholesterol reversal. The protocol details are presented in the Supporting Information.

### 2.12 RNA immunoprecipitation assay

RNA immunoprecipitation assays were performed by PureBinding RNA immunoprecipitation kit (Geneseed) to evaluate the interaction between the RPL22 protein and monocyte chemoattractant protein‐1 (*MCP‐1*) RNA in mouse BMDMs. In brief, 1 × 10^7^ cells were homogenised in immunoprecipitation lysis buffer containing RNasin and protease inhibitor, before being split into two samples for anti‐RPL22 and anti‐immunoglobulin G (IgG) treatment. Protein A/G Magnetic Beads were used to link the ribosomal protein RPL22 mouse monoclonal antibody (Santa Cruz) or IgG as the negative control, followed by incubation with cell lysis buffer to capture RNA–protein complexes overnight at 4°C. The relative enrichment of human or mouse *MCP‐1* mRNA was analysed by qRT‐PCR. The protocol details are presented in the Supporting Information.

### 2.13 qRT‐PCR and western blot

mRNA expression was analysed by qRT‐PCR using the ABI 7500 System (Applied Biosystems), and the primers are shown in Table S3. The protein expression of RPL22 and MCP‐1 was determined by western blotting. The protocol details are presented in the Supporting Information.

### 2.14 Statistical analysis

All experimental data were tested for normality using the Shapiro–Wilk normality test. For normally distributed data, the two‐tailed Student's *t*‐test was used for comparisons between two groups, and one‐way analysis of variance with Tukey's multiple‐comparisons test was used for multiple‐group comparisons. For non‐normally distributed data, the Mann–Whitney *U*‐test or the Kruskal–Wallis test followed by Dunn's multiple‐comparison test was performed, as appropriate. Power analyses were conducted with G*Power software (version 3.1.9.7) to calculate the statistical power based on the current sample size. In the discovery cohort, with an α level of  .05 and an effect size (*d*) of 1.60, the statistical power was  .748. In the validation cohort, with an effect size (*d*) of 1.50 for group comparisons, the statistical power was  .998.

SPSS (version 20.0; SPSS Inc.) was used for the statistical analyses. Two‐sided *p* < .05 was considered statistically significant. Technical repeats and single mice are indicated using symbols, where appropriate. All data are presented as the mean ± standard error of the mean.

## 3 RESULTS

### 3.1 *LncAPAT* is highly expressed in the peripheral blood of patients with coronary plaques

**FIGURE 1:**
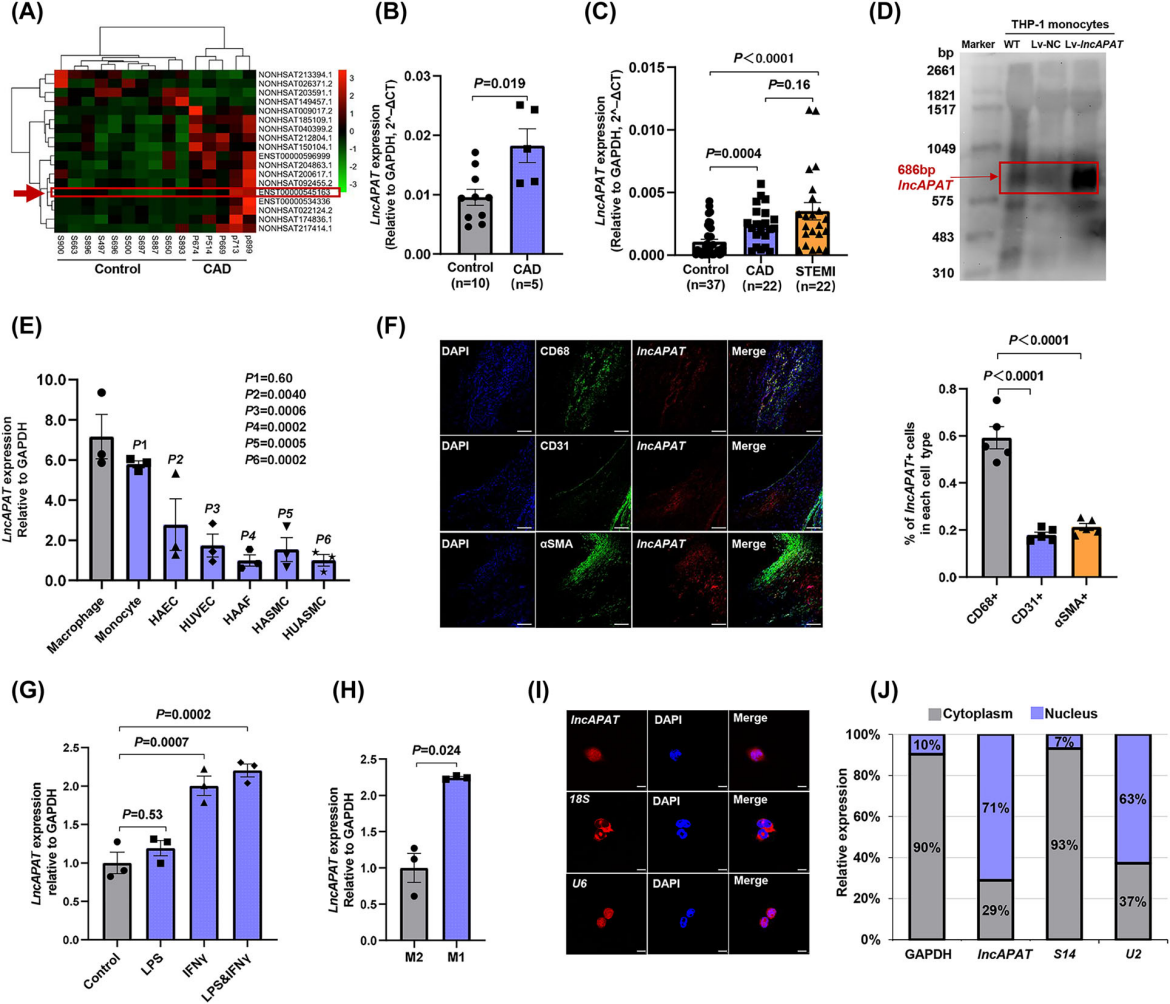
*LncAPAT* is highly expressed in the peripheral blood of patients with coronary mixed plaques. (A) Heatmap of differentially expressed transcripts in peripheral blood of patients with coronary artery disease (CAD) with mixed plaques on coronary computed tomography angiography (CCTA; *n* = 5) compared with control subjects (*n* = 10). (B) The expression of *lncAPAT* was determined by the quantitative real‐time polymerase chain reaction (qRT‐PCR) method in the same samples. (C) The expression of *lncAPAT* was determined by the qRT‐PCR method in the peripheral blood of CAD patients with CCTA examination (*n* = 22), acute ST‐elevation myocardial infarction (STEMI) patients with optical coherence tomography examination (*n* = 22), and control subjects (*n* = 37). (D) Northern blotting analysis of *lncAPAT* expression in THP‐1 monocytes. (E) The expression profiling of *lncAPAT* in vascular cells, including THP‐1 macrophages and monocytes, human aortic endothelial cells (HAECs), human umbilical vein endothelial cells (HUVECs), human aortic adventitial fibroblast cells (HAAFs), human aortic smooth muscle cells (HASMCs), and human umbilical artery smooth muscle cells (HUASMCs; *n* = 3 in each group). (F) Representative images and quantification of fluorescence in situ hybridisation for *lncAPAT* (red) and immunostaining for CD68 (green), CD31 (green), αSMA (green), along with 4′,6‐diamidino‐2‐phenylindole (DAPI) nuclear stain (blue) in carotid atherosclerotic plaques from patients undergoing carotid endarterectomy surgery (*n* = 5). Scale bar = 250 µm. (G) The expression levels of *lncAPAT* in THP‐1 monocytes when treated with LPS, IFNγ, and LPS & IFNγ, respectively. (H) The expression of *lncAPAT* in M1 and M2 macrophages derived from THP‐1 monocytes (*n* = 3 in each group). (I) RNA fluorescence in situ hybridisation for *lncAPAT* was performed in macrophages derived from THP‐1 monocytes, and *18S* ribosomal RNA and *U6* small nuclear RNA were used as positive controls of cytoplasm and nucleus, respectively. Scale bar = 8 µm. (J) Quantitative analysis of *lncAPAT* in cytoplasm and nucleus of macrophages derived from THP‐1 monocytes by qRT‐PCR method, and ribosomal protein *S14* and *U2* small nuclear ribonucleoprotein were used as endogenous controls of cytoplasm and nucleus, respectively (*n* = 3 in each group). For B and C, grey bars represent control subjects, blue bars represent CAD patients, and orange bars represent STEMI patients. For E, G and H, grey bars represent control group, blue bars represent experimental group. For F, grey bars represent CD68⁺ macrophages, blue bars represent CD31⁺ endothelial cells, and orange bars represent α‐SMA⁺ smooth muscle cells. For I, grey bars represent cytoplasm, blue bars represent nucleus. Values within the bar represent the percentage of *lncAPAT* expression. Mann–Whitney *U*‐test was used for B. Kruskal–Wallis test followed by Dunn's multiple‐comparison tests was used for C. One‐way ANOVA with Tukey's multiple‐comparisons test was used for E, F, and G. The two‐tailed unpaired *t*‐test was used for H. Data are mean ± standard error of the mean (SEM).

To screen differentially expressed *lncRNAs* associated with atherosclerotic plaque instability, whole‐transcriptome sequencing was performed using peripheral blood samples from control individuals and from CAD patients with mixed plaques on CCTA. A total of 18 *lncRNAs* were found to be differentially expressed with a cutoff threshold of fold change ≥2, false discovery rate ≤.01 and FPKM ≥1 (Figure 1A), and subsequently verified by qRT‐PCR analysis (Figure S2). Among these transcripts, a novel *lncRNA* transcript, poly (ADP‐ribose) polymerase family member 11 antisense RNA 1 202 (Ensembl ID: ENST00000545163), located on chromosome 12 with a length of 686 bp, was particularly notable because of the high expression in patients with mixed coronary plaques. This transcript was named *lncAPAT* (Figure 1B). Agarose gel electrophoresis was also performed to evaluate the RT‐PCR product yield of *lncAPAT* in peripheral blood RNA samples from these participants (Figure S3). Furthermore, in a validation cohort comprising 81 subjects (37 control subjects, 22 CAD patients with mixed plaque, and 22 patients with acute STEMI), *lncAPAT* expression in peripheral blood RNA samples was elevated by 2.7‐fold in patients with CAD and by 2.9‐fold in patients with STEMI compared with the control individuals, respectively. No significant change in *lncAPAT* expression was observed between CAD and STEMI patients (Figure 1C).

Considering the heterogeneity of peripheral blood, magnetic bead‐based separation was used to isolate lymphocytes (CD19⁺ B cells and CD3⁺ T cells) and CD14⁺ monocytes/macrophages from the peripheral blood of four control subjects to assess the cell‐type specificity of *lncAPAT* expression. The results show that *lncAPAT* was highly expressed in CD3^+^ T cells (Figure S4). We focused on macrophages owing to their predominance within atherosclerotic plaques and their essential functions across all stages of atherosclerosis, ranging from lesion initiation to regression.^14^, ^15^ Furthermore, northern blot analysis confirmed the expression of *lncAPAT* in human acute monocytic leukaemia cell line (THP‐1) monocytes (Figure 1D). *LncAPAT* expression profiling was conducted in vascular cells, including THP‐1 monocytes/macrophages, endothelial cells, smooth muscle cells, and aortic adventitial fibroblasts. The data show that *lncAPAT* was highly expressed in monocytes and macrophages (Figure 1E). Combined immunofluorescence and fluorescence in situ hybridisation analyses of carotid atherosclerotic plaques from patients undergoing carotid endarterectomy surgery showed that *lncAPAT* expression was significantly higher in macrophages than in smooth muscle cells and endothelial cells (Figure 1F).

Given that atherosclerosis is a chronic inflammatory disorder, THP‐1 monocytes were treated with several proinflammatory cytokines (lipopolysaccharide [LPS], interferon gamma [IFN‐γ], and LPS + IFN‐γ) to mimic the in vivo inflammatory microenvironment. IFN‐γ alone significantly increased *lncAPAT* expression by 2.0‐fold compared with the control group. Moreover, co‐stimulation with LPS and IFN‐γ further enhanced *lncAPAT* expression to about 2.2‐fold (Figure 1G). The expression of *lncAPAT* was 2.2‐fold higher in proinflammatory macrophages (M1) than in anti‐inflammatory macrophages (M2) (Figure 1H). RNA fluorescence in situ hybridisation and cytoplasmic and nuclear RNA purification assays were used to determine the subcellular localisation of *lncAPAT* in macrophages. The results showed that *lncAPAT* was distributed in both the nucleus and the cytoplasm (Figure 1I), but it predominated in the nucleus (71%; Figure 1J).

### 3.2 *LncAPAT* promotes atherosclerotic plaque formation in vivo

A phylogenetic tree of *lncAPAT* was constructed, and no homologous sequence of *lncAPAT* was detected in mice (Figure S5). Conservation of *lncAPAT* between humans and mice was not observed in either the NCBI (https://www.ncbi.nlm.nih.gov/) or UCSC (http://genome.ucsc.edu/) databases. To explore the effects of *lncAPAT* on atherosclerotic lesions in vivo, the myeloid cell‐specific *lncAPAT* knock‐in mouse model was established (Figure S6A). All mice were genotyped (Figure S6B), and *lncAPAT* expression was validated in peritoneal macrophages, PBMCs, and BMDMs by qRT‐PCR. Along with monocytes and macrophages, *lncAPAT* was also overexpressed in neutrophils (Figure S6C). Combined immunofluorescence and fluorescence in situ hybridisation analyses of thoracic aortic plaques from *lncAPAT^flox/flox^;* *LyzM*‐Cre^+/−^mice showed that *lncAPAT* expression level was comparable between neutrophils and macrophages within atherosclerotic plaques, and among the immune cells that infiltrated atherosclerotic plaques, macrophages were the predominant cell type (Figure S6D).

At baseline, *lncAPAT^flox/flox^;LyzM*‐Cre^+/−^ and *lncAPAT^flox/flox^* mice showed no significant differences in serum lipid profiles, transaminase activities, and glucose levels. Peripheral blood immune cell composition, including neutrophils, lymphocytes, monocytes, eosinophils, and basophils, was also comparable between the two groups (Figure S7A,B). Histological analysis of thoracic aortas further revealed similar aortic wall thickness and collagen content in both genotypes, indicating no pre‐existing structural or compositional differences in the vasculature (Figure S7C). In addition, to exclude potential effects of low‐level basal transcription of *lncAPAT* on baseline phenotypes, *lncAPAT^flox/flox^* were compared with wild‐type mice under basal conditions. No significant differences were observed between the two groups in serum laboratory parameters, immune cell composition, and thoracic aortic morphology (Figure S7).

**FIGURE 2:**
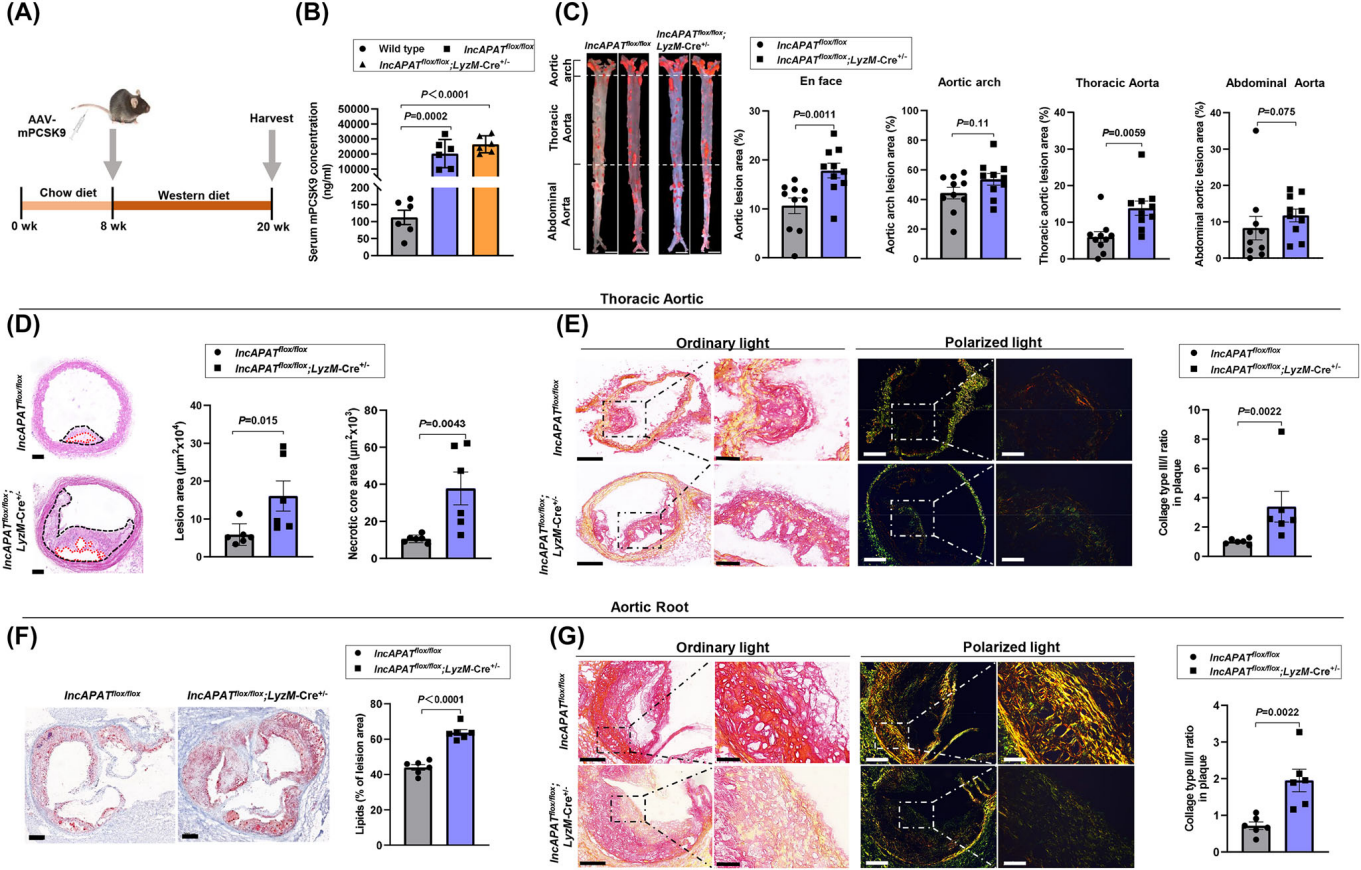
*LncAPAT* promotes the formation of atherosclerotic plaques in vivo. (A) The schema of mouse model establishment: the *lncAPAT^flox/flox^* and *lncAPAT^flox/flox^;LyzM*‐Cre^+/−^ mice were injected with a recombinant adeno‐associated virus of murine PCSK9 (rAAV‐mPCSK9) and then fed with Western diet for 12 weeks to induce atherosclerosis lesions. (B) Serum levels of mPCSK9 in mice injected with saline or rAAV‐mPCSK9 (*n* = 6 in each group). (C) Representative images of en face analysis and quantification of atherosclerosis lesion areas in the whole aorta, aortic arch, thoracic aorta, and abdominal aorta (*n* = 10 in each group). Scale bar = 500 µm. (D) Representative images of haematoxylin–eosin staining and quantification of atherosclerosis lesion areas and necrotic core areas in cross sections of the thoracic aorta (*n* = 6 in each group). Scale bar = 100 µm. (E) Representative images of picrosirius red staining and quantification of the ratio of type III/type I collagen in atherosclerosis lesion areas of cross sections of the thoracic aorta (*n* = 6 in each group). Narrowed view, scale bar = 200 µm; enlarged view, scale bar = 50 µm. (F) Representative images of Oil red O staining and quantification of lipid deposition in atherosclerotic lesion areas of the aortic valve (*n* = 6 in each group). Scale bar = 200 µm. (G) Representative images of picrosirius red staining and quantification of the ratio of type III/type I collagen in atherosclerosis lesion areas of the aortic valve (*n* = 6 in each group). Narrowed view, scale bar = 200 µm; enlarged view, scale bar = 50 µm. For B, grey bars represent wild‐type mice, blue bars represent *lncAPAT^flox/flox^* mice and orange bars represent *lncAPAT^flox/flox^;* *LyzM*‐Cre^+/−^ mice. For C through G, grey bars represent *lncAPAT^flox/flox^* mice, blue bars represent *lncAPAT^flox/flox^;* *LyzM*‐Cre^+/−^ mice. One‐way analysis of variance (ANOVA) with Tukey's multiple‐comparisons test was used for B. Mann–Whitney *U*‐test was used for C through G. Data are mean ± standard error of the mean (SEM).

*LncAPAT^flox/flo^* ^x^ and *lncAPAT^flox/flo^* ^x^ *;* *LyzM*‐Cre^+/−^ mice were injected with rAAV‐mPCSK9 and fed a western diet for 12 weeks to induce atherosclerotic lesions (Figure 2A). Serum mPCSK9 was measured, confirming successful model establishment (Figure 2B). Serum lipids, transaminase, glucose, and body weight were measured, and no differences were observed between *lncAPAT^flox/flox^;LyzM*‐Cre^+/−^ and *lncAPAT^flox/flox^* mice (Figure S8).

The analysis of atherosclerosis lesion area in en face aortae revealed that atherosclerotic lesion formation was increased by 1.7‐fold in *lncAPAT^flox/flox^;LyzM*‐Cre^+/−^ mice compared with *lncAPAT^flox/flox^* mice (Figure 2C). Further analysis showed a remarkable increase in the thoracic aortic area of plaque lesions (Figure 2C), in which the absolute lesion area and necrotic core increased by 2.7‐fold and 3.6‐fold, respectively (Figure 2D), and the ratio of type III/type I collagen increased by 3.5‐fold (Figure 2E). Compared with the control group, *lncAPAT* did not significantly alter collagen or lipid content either in the whole plaque or in non‐necrotic core regions (Figure S9A,B). In addition, no significant differences were observed in fibrous cap thickness or the number of broken elastic laminae in the aorta between the two groups (Figure S9C,D). In the aortic root area, lipid deposition and the ratio of type III/type I collagen were increased by 1.5‐fold and 2.7‐fold, respectively (Figure 2F,G), but the lesion area did not change significantly in *lncAPAT^flox/flox^;LyzM*‐Cre^+/−^ mice (Figure S10). The atherosclerotic plaque area was also measured in female *lncAPAT^flox/flox^;LyzM*‐Cre^+/−^ mice, and the data show that female *lncAPAT^flox/flox^;LyzM*‐Cre^+/−^ mice had a 3.0‐fold increase in atherosclerotic lesion area in the aortic artery compared with the control group (Figure S11).

### 3.3 *LncAPAT* promotes the inflammatory response of monocytes and macrophages in mice in vivo

**FIGURE 3:**
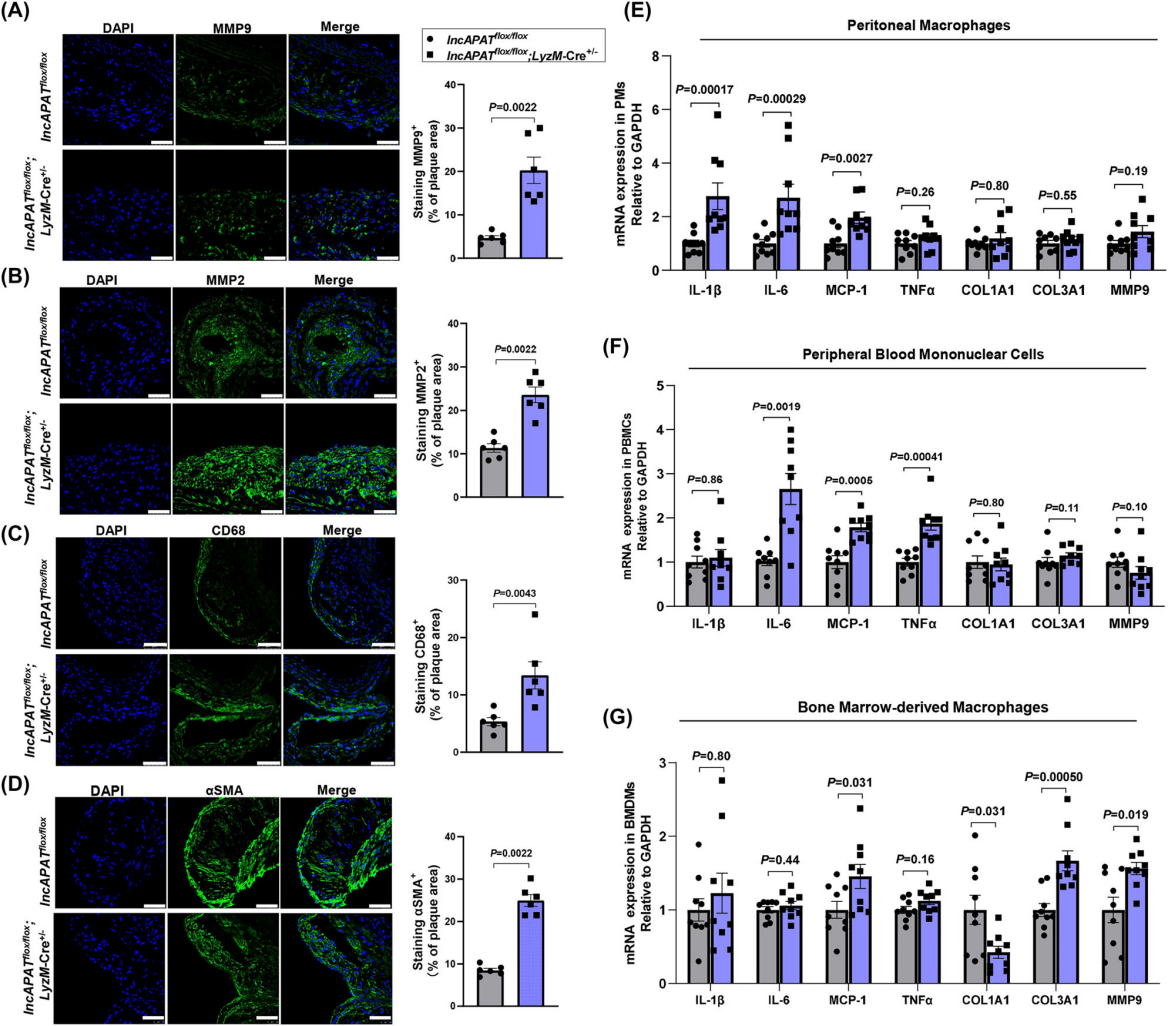
*LncAPAT* promotes the inflammatory response of monocytes in mice. (A) Representative images and quantification of immunostaining for MMP9 (green) and 4′,6‐diamidino‐2‐phenylindole (DAPI) nuclear stain (blue) in thoracic aorta plaques of *lncAPAT^flox/flox^* and *lncAPAT^flox/flox^;LyzM*‐Cre^+/−^mice (*n* = 6 in each group). Scale bar = 50 µm. (B) Representative images and quantification of immunostaining for MMP2 (green) and DAPI nuclear stain (blue) in thoracic aorta plaques of mice (*n* = 6 in each group). Scale bar = 50 µm. (C) Representative images and quantification of immunostaining for CD68 molecule (green) and DAPI nuclear stain (blue) in thoracic aorta plaques of mice (*n* = 6 in each group). Scale bar = 75 µm. (D) Representative images and quantification of immunostaining for αSMA (green) and DAPI nuclear stain (blue) in thoracic aorta plaques of mice (*n* = 6 in each group). Scale bar = 50 µm. E‐G, The mRNA expression of *IL‐1β*, *IL‐6*, monocyte chemoattractant protein‐1 (*MCP‐1*), tumour necrosis factor alpha (*TNFα*), *COL1A1*, *COL3A1*, and *MMP9* in peritoneal macrophages (E), peripheral blood mononuclear cells (PBMCs) (F), and bone marrow‐derived macrophages (BMDMs) (G) from mice (*n* = 9 in each group). Grey bars represent *lncAPAT^flox/flox^* mice, blue bars represent *lncAPAT^flox/flox^;LyzM*‐Cre^+/−^ mice. Mann–Whitney *U*‐test was used for A through G. Data are mean ± standard error of the mean (SEM).

MMPs play a crucial role in regulating inflammation by breaking down extracellular matrix components, which allows immune cells, such as monocytes, to migrate to the site of inflammation. To explore the effects of *lncAPAT* on the inflammatory response in vivo, immunofluorescence staining of MMPs was conducted in plaques from *lncAPAT^flox/flox^;LyzM*‐Cre^+/−^ and *lncAPAT^flox/flox^* mice. The data show that *lncAPAT* increased MMP9 expression by 4.3‐fold (Figure 3A) and MMP2 expression by 2.1‐fold (Figure 3B) in thoracic aortic plaques from *lncAPAT^flox/flox^;* *LyzM*‐Cre^+/−^ mice compared with *lncAPAT^flox/flox^* mice. *LncAPAT* also increased the number of macrophages and smooth muscle cells by 2.7‐fold (Figure 3C) and 2.0‐fold (Figure 3D), respectively, in thoracic aortic plaques from *lncAPAT^flox/flox^;LyzM*‐Cre^+/−^ mice.

Three monocyte and macrophage subpopulations (peritoneal macrophages, PBMCs, and BMDMs) isolated from *lncAPAT^flox/flox^* and *lncAPAT^flox/flox^;LyzM*‐Cre^+/−^ mice were analysed to determine whether the effects of *lncAPAT* were subset‐specific or broadly conserved across the myeloid lineage. Given the functional heterogeneity of monocyte‐derived cells in atherosclerosis, including their roles in inflammation, lipid handling, and tissue remodelling, characterising each subset provides mechanistic insights into how *lncAPAT* may contribute to atherosclerosis progression.

In peritoneal macrophages, *lncAPAT* significantly increased the mRNA expression of interleukin (*IL*)*‐1β*, *IL‐6* and *MCP‐1* (Figure 3E). In PBMCs, *lncAPAT* significantly increased the mRNA expression of *IL‐6*, tumour necrosis factor alpha (*TNFα*), and *MCP‐1* (Figure 3F). In BMDMs, *lncAPAT* significantly increased the mRNA expression of *MCP‐1*. The expression of *MMP9* and collagen type III alpha 1 chain (*COL3A1*) was increased, while the expression of collagen type I alpha 1 chain (*COL1A1*), which encodes a type of stable collagen, was decreased (Figure 3G). These data indicate that *lncAPAT* contributes to the inflammatory response in the context of atherosclerosis progression in mice.

### 3.4 *LncAPAT* promotes the inflammatory responses of monocytes, macrophages, endothelial cells, and smooth muscle cells in vitro

**FIGURE 4:**
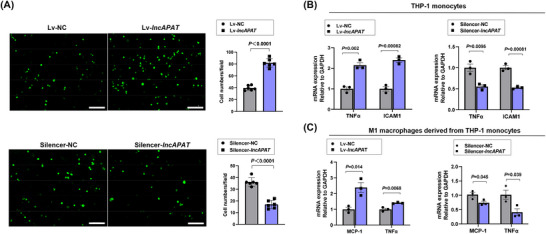
*LncAPAT* promotes the inflammatory response of macrophages in vitro. (A) Representative images and quantification of THP‐1 monocytes adhering to endothelial cells (*n* = 6 in each group). Scale bar = 200 µm. (B) The mRNA expression of tumour necrosis factor alpha (*TNFα*) and *ICAM‐1* in THP‐1 monocytes with overexpression or knockdown of *lncAPAT* (*n* = 3 in each group). (C) The mRNA expression of monocyte chemoattractant protein‐1 (*MCP‐1*) and *TNFα* in macrophages derived from THP‐1 monocytes with overexpression or knockdown of *lncAPAT* (*n* = 3 in each group). Grey bars represent the control group, blue bars represent the experimental group. The two‐tailed unpaired Student *t*‐test was used for A through C. Data are mean ± standard error of the mean (SEM).

To investigate the effects of *lncAPAT* on the inflammatory responses of monocytes and macrophages, THP‐1 cell lines stably overexpressing *lncAPAT* were established by lentiviral infection. In THP‐1 monocytes, a 2.1‐fold increase in the adhesion ability to endothelial cells was observed upon *lncAPAT* overexpression in the adhesion assays. The adhesion ability of monocytes was significantly inhibited by *lncAPAT* knockdown (Figure 4A). Furthermore, in THP‐1 monocytes, *lncAPAT* overexpression significantly increased the expression of the cytokines *TNFα* and intercellular adhesion molecule 1 (*ICAM‐1*), and their expression was decreased after *lncAPAT* knockdown (Figure 4B). In M1 macrophages derived from THP‐1 cells, *lncAPAT* increased the mRNA expression of *MCP‐1* and *TNFα*, whereas *lncAPAT* knockdown decreased their expression (Figure 4C). These results indicate that *lncAPAT* promotes the inflammatory response of monocytes and macrophages.

Given the critical roles of endothelial cells and smooth muscle cells in atherosclerosis progression, the function of *lncAPAT* in human umbilical vein endothelial cells (HUVECs) and human aortic smooth muscle cells (HASMCs) was investigated. In HUVECs, *lncAPAT* overexpression significantly increased the expression of the proinflammatory cytokines *MCP‐1*, *IL‐1β*, *IL‐6*, and *TNFα*, whereas *lncAPAT* knockdown decreased their expression (Figure S12A,B). Moreover, adhesion assays revealed that *lncAPAT* overexpression enhanced the adhesion ability of monocytes to endothelial cells by 3.0‐fold, which was inhibited by 49% after *lncAPAT* knockdown (Figure S12C). In HASMCs, *lncAPAT* overexpression significantly increased the expression of the proinflammatory cytokine *TNFα* and the phenotypic modulation‐associated cytokine *SM22α*, whereas *lncAPAT* knockdown decreased their expression (Figure S13A,B). Nevertheless, *lncAPAT* had no effect on the proliferation and migration of HUVECs and HASMCs (Figures S12D,E and S13C,D). These results suggest that *lncAPAT* may participate in atherosclerosis by modulating the inflammatory responses of various cell types, including macrophages, endothelial cells, and smooth muscle cells.

### 3.5 *LncAPAT* promotes cholesterol accumulation and inhibits cholesterol efflux in macrophages in vitro and in vivo

**FIGURE 5:**
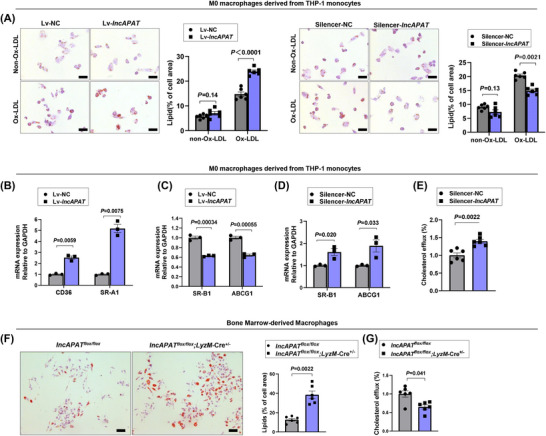
*LncAPAT* promotes cholesterol accumulation and inhibits cholesterol reversal of macrophages in vitro and in vivo. (A) Representative images and quantification of cholesterol accumulation in macrophages derived from THP‐1 monocytes incubated with or without oxidised low‐density lipoprotein, as detected by Oil red O staining (*n* = 6 in each group). Scale bar = 50 µm. (B) The mRNA expression of lipid phagocytosis‐related genes *CD36* molecule and S*R‐A1* in macrophages derived from THP‐1 monocytes with overexpression of *lncAPAT* by lentivirus (*n* = 3 in each group). (C, D) The mRNA expression of cholesterol reversal‐related genes *SR‐B1* and *ABCG1* in macrophages derived from THP‐1 monocytes with overexpression of *lncAPAT* by lentivirus (*n* = 3 in each group) (C) or knockdown of *lncAPAT* by smart silencer (*n* = 3 in each group) (D). (E) The quantification of cholesterol reversal in macrophages derived from THP‐1 monocytes with knockdown of *lncAPAT* by smart silencer (*n* = 6 in each group). (F) Representative images and quantification of cholesterol accumulation in bone marrow‐derived macrophages (BMDMs) isolated from *lncAPAT^flox/flox^* and *lncAPAT^flox/flox^;LyzM*‐Cre^+/−^ mice incubated with oxidised low‐density lipoprotein, as detected by Oil red O staining (*n* = 6 in each group). Scale bar = 50 µm. (G) The quantification of cholesterol efflux in BMDMs isolated from *lncAPAT^flox/flox^* and *lncAPAT^flox/flox^;LyzM*‐Cre^+/−^ mice by cholesterol efflux assay (*n* = 6 in each group). Grey bars represent the control group, blue bars represent the experimental group. The two‐tailed unpaired Student *t*‐test was used for A through E. Mann–Whitney *U*‐test was used for F and G. Data are mean ± standard error of the mean (SEM).

Under basal conditions, *lncAPAT* did not affect macrophage cholesterol accumulation. Upon exogenous ox‐LDL stimulation, *lncAPAT* overexpression increased cholesterol accumulation by 98%, which was inhibited by 72% after *lncAPAT* knockdown (Figure 5A). *LncAPAT* increased the expression of the lipid phagocytosis‐related genes *CD36* by 2.5‐fold and the expression of scavenger receptor A1 by 5.2‐fold (Figure 5B), while the expression of cholesterol efflux‐related genes, including the gene encoding scavenger receptor B1 (*SR‐B1*) and ATP‐binding cassette subfamily G member 1 (*ABCG1*), was inhibited by 38% and 36%, respectively (Figure 5C). Correspondingly, the expression of *SR‐B1* and *ABCG1* mRNA (Figure 5D) and the cholesterol efflux ratio (Figure 5E) were increased after *lncAPAT* knockdown. In BMDMs isolated from *lncAPAT^flox/flox^;LyzM*‐Cre^+/−^ mice, *lncAPAT* knock‐in increased cholesterol accumulation in macrophages by 3.0‐fold (Figure 5F) and reduced the cholesterol efflux ratio of macrophages by 35% compared with that observed in *lncAPAT^flox/flox^* mice (Figure 5G).

### 3.6 *LncAPAT* binds with the promoter region of *RPL22*

**FIGURE 6:**
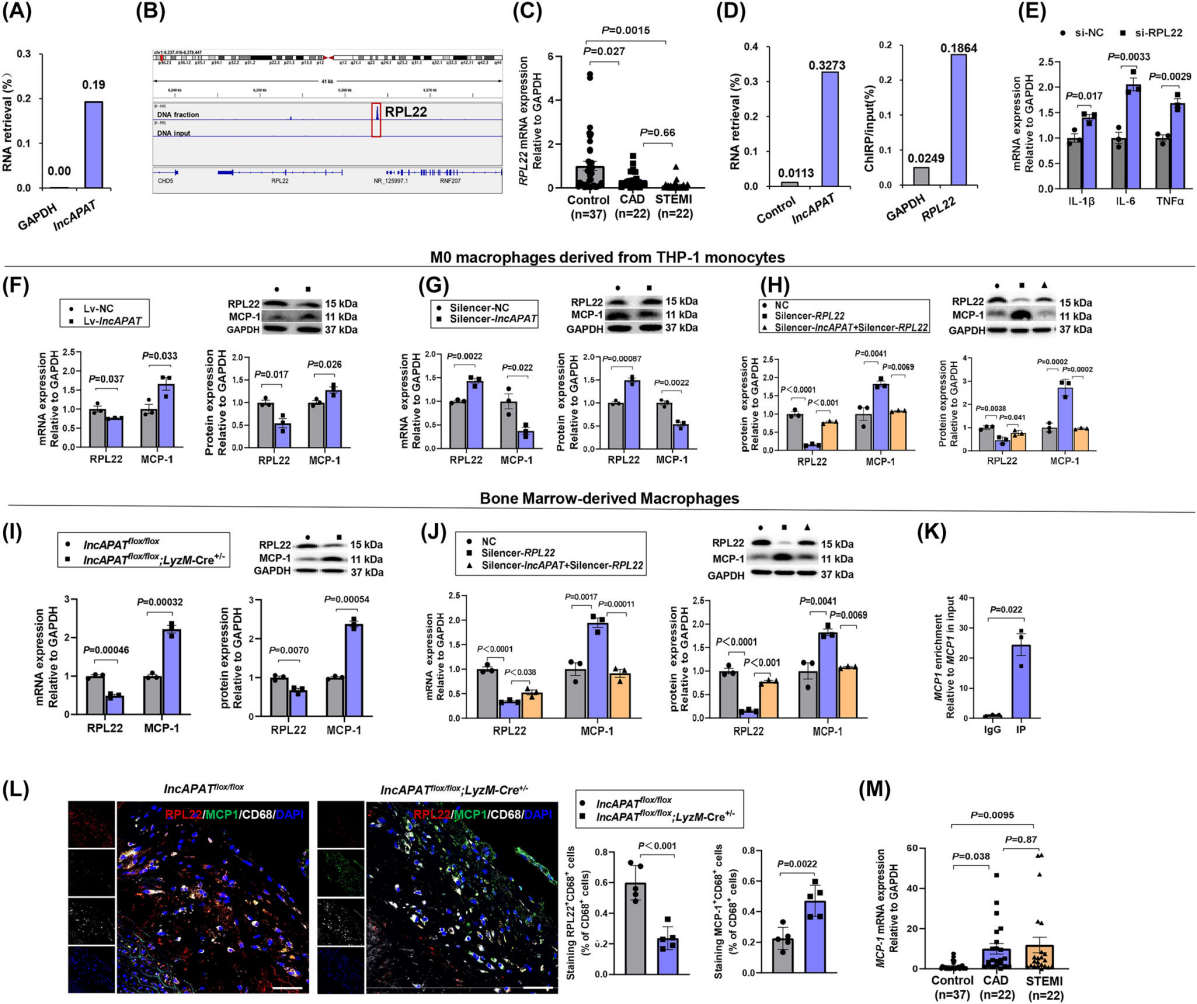
*LncAPAT* regulates the inflammatory response of macrophages by targeting *RPL22*. (A) Relative *lncAPAT* retrieval in THP‐1‐derived macrophages by chromatin isolation by RNA purification (ChIRP) assay (*n* = 2 in each group). (B) ChIRP‐Seq analysis of *lncAPAT* binding at the *RPL22* gene locus in the DNA input and fraction sample. (C) The mRNA expression of *RPL22* was determined by the quantitative real‐time polymerase chain reaction (qRT‐PCR) method in the peripheral blood of coronary artery disease (CAD) patients with coronary computed tomography angiography (CCTA) examination (*n* = 22), acute ST‐elevation myocardial infarction (STEMI) patients with optical coherence tomography examination (*n* = 22), and control subjects (*n* = 37). (D) Relative *lncAPAT* retrieval by ChIRP assay and the expression of *GAPDH* and *RPL22* fracted with *lncAPAT* probe from bone marrow‐derived macrophages (BMDMs) of *lncAPAT^flox/flox^;LyzM*‐Cre^+/−^ mice (*n* = 2 in each group). (E) The mRNA expression of *IL‐1β, IL‐6*, and tumour necrosis factor alpha (*TNFα*) in macrophages derived from THP‐1 monocytes with knockdown of *RPL22* by smart silencer (*n* = 3 in each group). (F) The mRNA and protein levels of *RPL22* and monocyte chemoattractant protein‐1 (*MCP‐1*) in THP‐1 monocytes with overexpression of *lncAPAT* by lentivirus (*n* = 3 in each group). (G) The mRNA and protein levels of *RPL22* and *MCP‐1* in THP‐1 monocytes with knocking down of *lncAPAT* by smart silencer (*n* = 3 in each group). (H) The mRNA and protein levels of *RPL22* and *MCP‐1* in THP‐1 monocytes with knocking down of *RPL22* alone or knocking down of *RPL22* and *lncAPAT* together to verify the reverse effects of *RPL22* on inflammation induced by *lncAPAT* (*n* = 3 in each group). (I) The mRNA and protein levels of *RPL22* and *MCP‐1* in BMDMs from *lncAPAT^flox/flox^* and *lncAPAT^flox/flox^;LyzM*‐Cre^+/−^ mice (*n* = 3 in each group). (J) The mRNA and protein levels of *RPL22* and *MCP‐1* in BMDMs from *lncAPAT^flox/flox^;LyzM*‐Cre^+/−^ mice with further knocking down of *RPL22* alone or knocking down of *RPL22* and *lncAPAT* together to clarify the reverse effects of *RPL22* on inflammation induced by *lncAPAT* (*n* = 3 in each group). (K) Relative enrichment of *MCP‐1* in BMDMs by RNA immunoprecipitation assay. (L) Representative images and quantification of immunostaining for RPL22 (red), MCP‐1 (green), CD68 molecule (white), and 4′,6‐diamidino‐2‐phenylindole (DAPI) nuclear stain (blue) in thoracic aorta plaques of *lncAPAT^flox/flox^* and *lncAPAT^flox/flox^;LyzM*‐Cre^+/−^ mice (*n* = 5 in each group). Scale bar = 20 µm. (M) The mRNA expression of *MCP‐1* in CAD patients with CCTA examination (*n* = 22), acute ST‐elevation myocardial infarction (STEMI) patients with optical coherence tomography examination (*n* = 22), compared with control subjects (*n* = 37). For A and D through L, grey bars represent the control group, blue or orange bars represent the experimental group. For C and M, grey bars represent control subjects, blue bars represent CAD patients and orange bars represent STEMI patients. Kruskal–Wallis test followed by Dunn’s multiple‐comparison tests was used for C and M. The two‐tailed unpaired Student *t*‐test was used for E, F, G, I, and K. One‐way analysis of variance (ANOVA) with Tukey’s multiple‐comparisons test was used for H and J. Mann–Whitney *U*‐test was used for L. Data are mean ± standard error of the mean (SEM).

To explore the molecular mechanism by which *lncAPAT* regulates macrophage inflammation and atherosclerotic plaque instability, potential targets of *lncAPAT* were identified by the ChIRP assay and sequencing. In the ChIRP assay, the RNA retrieved by *lncAPAT* probes was quantified by qRT‐PCR (Figures 6A and S14A). ChIRP‐Seq was performed twice to identify the potential targets of *lncAPAT*. Approximately 30 000 and 23 000 peaks were enriched from the macrophage genome. The intersection of the peaks from two ChIRP sequences revealed 563 genes. Five genes (*AK8*, *SNX16*, *PTBP1*, *RNF207*, and *RPL22*) had promoters that were directly bound with *lncAPAT* after filtering out pseudogenes, non‐coding RNAs, and unannotated genes. The annotations of *AK8*, *SNX16*, *PTBP1*, *RNF207*, and *RPL22* are provided in Table S4. The direct binding of *lncAPAT* with the promoter of *RPL22* from the ChIRP‐Seq analysis is shown in Figure 6B. We quantified the mRNA levels of these five genes in peripheral blood samples from the indicated cohort. Compared with control subjects, *RPL22* expression was significantly reduced by 65% in CAD and 92% in STEMI patients, but showed no marked difference between the two patient groups (Figure 6C). Conversely, the expression of *AK8, SNX16, PTBP1*, and *RNF207* remained unchanged across the control, CAD, and STEMI groups (Figure S15A–D). Furthermore, Spearman analysis revealed a significant negative correlation between *lncAPAT* and *RPL22* mRNA levels in our cohorts (*r* = −.4, *p* = .0002; Figure S15E). RPL22 is a component of the 60S ribosomal subunit assembled in the nucleolus, and the promoter region of *RPL22* gene contains four putative NF‐κB binding domains, which indicates its potential role in inflammation and related diseases.^16^ Das et al. reported that RPL22 protein directly interacts with the 5′‐untranslated region of *MCP‐1* mRNA and thereby promotes its decay, a process considered to be critical for inflammation.^17^ In the present study, together with the results of the ChIRP assay and sequencing data, *RPL22* was selected as the potential target of *lncAPAT* for the subsequent studies, and *MCP‐1* was selected as the putative downstream target of *RPL22*.

To verify the interaction between *RPL22* and *lncAPAT*, we conducted the ChIRP assay followed by qRT‐PCR of BMDMs from *lncAPAT^flox/flox^;* *LyzM*‐Cre^+/−^ mice. The results showed that the content of *lncAPAT* enriched by the *lncAPAT* probe was 27.9‐fold higher than in the control group. The content of *RPL22* pulled down by the *lncAPAT* probe was 6.5‐fold higher than the internal reference (*GAPDH*, encoding glyceraldehyde‐3‐phosphate dehydrogenase; Figures 6D and S14B,C), suggesting the direct binding of *lncAPAT* to *RPL22*. Furthermore, we knocked down the mRNA expression of *RPL22* in THP‐1‐derived macrophages using a smart silencer to assess its direct role in regulating the macrophage inflammatory response. *RPL22* inhibition significantly increased the mRNA expression of inflammatory cytokines (*IL‐1β* by 1.4‐fold, *IL‐6* by 2.1‐fold, and *TNFα* by 1.7‐fold), indicating that *RPL22* knockdown alone induced a proinflammatory response similar to that observed with *lncAPAT* overexpression (Figure 6E).

Experiments on macrophages derived from THP‐1 monocytes showed that *lncAPAT* overexpression significantly inhibited the expression of *RPL22* and subsequently increased the expression of the inflammatory cytokine *MCP‐1*, both at the mRNA and protein levels (Figure 6F). In contrast, after *lncAPAT* knockdown from macrophages, the mRNA and protein expression of *RPL22* was significantly increased by 1.4‐fold and 1.5‐fold, respectively, and the mRNA and protein expression of *MCP‐1* was inhibited by 63% and 46%, respectively (Figure 6G).

To further investigate whether *MCP‐1* is regulated by *RPL22*, we silenced *RPL22* mRNA expression in THP‐1‐derived macrophages. *RPL22* silencing led to a 1.6‐fold and 3.7‐fold increase in *MCP‐1* mRNA and protein expression, respectively (Figure 6H). The upregulation of *MCP‐1* mRNA and protein expression induced by *RPL22* silencing was blocked after knockdown of both *RPL22* and *lncAPAT* in THP‐1‐derived macrophages (Figure 6H), indicating that *lncAPAT* regulates the expression of the inflammatory factor *MCP‐1* through *RPL22*. To verify the interaction between *RPL22* and *lncAPAT*, we evaluated the expression of *RPL22* and *MCP‐1* in BMDMs from *lncAPAT^flox/flox^;* *LyzM*‐Cre^+/−^ mice. Knock‐in of *lncAPAT* significantly inhibited *RPL22* mRNA and protein expression by 51% and 33%, respectively, and increased the mRNA and protein expression of *MCP‐1* by 3.2‐fold and 3.4‐fold, respectively (Figure 6I). In addition, *RPL22* knockdown in BMDMs from *lncAPAT^flox/flox^;LyzM*‐Cre^+/−^ mice significantly increased *MCP‐1* mRNA and protein expression by 1.9‐fold and 1.8‐fold, respectively (Figure 6J). Knockdown of both *RPL22* and *lncAPAT* blocked the upregulation of *MCP‐1* in BMDMs from *lncAPAT^flox/flox^;* *LyzM*‐Cre^+/−^ mice (Figure 6J). RNA immunoprecipitation experiments were conducted using BMDMs from *lncAPAT^flox/flox^;LyzM*‐Cre^+/−^ mice to explore the interaction between *RPL22* and *MCP‐1*. The content of *MCP‐1* was enriched by the RPL22 antibody (24‐fold higher than the negative control group), suggesting direct binding between the RPL22 protein and *MCP‐1* mRNA (Figures 6K and S16). Immunofluorescence analysis revealed markedly reduced expression of RPL22, concomitantly with elevated MCP‐1 levels, in macrophages within the aortic plaques of *lncAPAT^flox/flox^;LyzM*‐Cre^+/−^ mice compared to *lncAPAT^flox/flox^* mice (Figure 6L). Consistently, in female mice with aortic plaques, *lncAPAT^flox/flox^;LyzM*‐Cre^+/−^ mice showed decreased *RPL22* and increased MCP‐1 expression (Figure S17). These findings indicate that *lncAPAT* modulates *RPL22* and *MCP‐1* in vivo. In the peripheral blood of CAD and STEMI patients, the mRNA expression of *MCP‐1* was 7.8‐fold and 9.2‐fold higher than that in control individuals, respectively (Figure 6M).

To determine whether the *lncAPAT–RPL22* axis regulates other inflammatory mediators beyond *MCP‐1*, we analysed *IL‐6*, *IL‐1β*, *TNFα*, *MMP‐2*, and *MMP‐9* expression in BMDMs from *lncAPAT^flox/flox^;LyzM*‐Cre^+/−^ mice under the conditions of Figure 6J. *RPL22* knockdown significantly upregulated the mRNA expression of *IL‐1β* and *TNFα*, and moderately increased *IL‐6*. However, concurrent knockdown of *lncAPAT* failed to rescue these expression changes (Figure S18). These data suggest that the *lncAPAT–RPL22* axis specifically and directly regulates *MCP‐1*, without broadly altering the inflammatory cytokine landscape. To further explore whether *lncAPAT* regulates other chemokines in macrophages, we assessed the mRNA levels of *CXCL9*, *CXCL10*, *CXCL11*, *CCL3*, and *CXCL4* in THP‐1–derived macrophages after *lncAPAT* overexpression or knockdown. None of these chemokines exhibited significant changes, supporting a relatively specific role of *lncAPAT* in modulating *MCP‐1*. (Figure S19).

Collectively, these data indicate that *lncAPAT* promoted the inflammatory response of macrophages by inhibiting the transcriptional activity of *RPL22* and selectively upregulating *MCP‐1*, thereby contributing to atherosclerotic plaque instability.

## 4 DISCUSSION

Macrophages play a pivotal role in the development of atherosclerotic lesions. We identified a novel *lncRNA*, namely, *lncAPAT*, which was highly expressed in the peripheral blood of patients with unstable coronary artery atherosclerotic plaques. In animal models of atherosclerosis, myeloid cell‐specific overexpression of *lncAPAT* enhanced the atherosclerotic plaque lesion area, increased the ratio of type III/type I collagen, and promoted the expression of *MMP9* and *MMP2* in aortic plaques. In PBMCs isolated from *lncAPAT^flox/flox^;LyzM*‐Cre^+/−^ mice, *lncAPAT* increased *IL‐6* and *TNFα* inflammatory cytokine expression. *LncAPAT* significantly promoted monocyte adhesion to the endothelium in vitro. Moreover, *lncAPAT* directly targeted the promoter region of *RPL22*, increased *MCP‐1* expression, and promoted the inflammatory response of macrophages by inhibiting the transcriptional activity of *RPL22. RPL22* expression in the peripheral blood of patients with mixed plaques was lower than in control individuals without coronary artery plaques. These findings indicate a novel molecular mechanism underpinning atherosclerotic plaque instability, in which *lncAPAT* regulates the inflammatory response of macrophages by targeting *RPL22*.

Atherosclerosis is characterised by vascular inflammatory lesions that exist in the coronary arteries and large blood vessels, such as the aorta (including the aortic sinus, aortic arch, thoracic aorta, and abdominal aorta). Plaque lesions at different vascular sites exhibit localisation‐dependent responses during atherosclerosis progression. Czamara et al. used fibreoptic Raman spectroscopy and Raman microscopy to explore changes in the aortic wall and adjacent perivascular adipose tissue in a murine model of atherosclerosis (*Apo^−/−^/Ldlr^−/−^* mice), revealing that the abdominal and thoracic segments of the aorta exhibit distinct chemical compositions and patterns of lipid deposition in the intimal–medial layers.^18^ Furthermore, Benvenuti et al. found that atherosclerosis progression was more rapid in the abdominal aorta than in the thoracic aorta.^19^ In the present study, we found that *lncAPAT* increased the size of plaque lesions at the en face aorta in the murine model of atherosclerosis induced by mPCSK9 (*lncAPAT^flox/flox^;LyzM*‐Cre^+/−^ mice), and the difference was more significant in the thoracic aorta than in the aortic arch and abdominal aorta. In the thoracic aorta, *lncAPAT* increased the lesion area and the ratio of type III/type I collagen, but the total collagen content and lipid deposition were not affected by *lncAPAT*. In the aortic sinus, *lncAPAT* promoted lipid deposition and increased the ratio of type III/type I collagen, but the lesion area was unchanged. One potential explanation could be that the influence of *lncAPAT* in atherosclerosis progression may vary depending on the aortic artery site. Type I collagen is considered to be a mature and mechanically strong component of the fibrous cap, whereas type III collagen is relatively immature and weaker. A higher type III/type I ratio indicates a softer, more disorganised extracellular matrix and thus reduced plaque stability.^20^, ^21^ These findings support that *lncAPAT* overexpression promotes a collagen subtype shift towards an unstable plaque composition, contributing to decreased plaque stability.

Atherosclerosis is a chronic inflammatory disorder characterised by the presence of immune cells, predominantly those that produce proinflammatory cytokines, in lesions. This continuous inflammatory response leads to endothelial cell activation, smooth muscle cell proliferation, lesion progression, and ultimately vulnerable plaque destabilization by matrix degradation of the fibrous cap. We observed that *lncAPAT* overexpression increased the expression of the inflammatory factor *TNFα* in THP‐1‐derived macrophages, while *lncAPAT* knockdown decreased *TNFα* expression. Moreover, in myeloid cell‐specific *lncAPAT* knock‐in mice (*lncAPAT^flox/flox^;LyzM*‐Cre^+/−^ mice), *lncAPAT* promoted *TNFα* expression in PBMCs. *TNFα* modulates leukocyte activation and maturation, cytokine and chemokine release, and the generation of reactive oxygen species and nitrogen intermediates in atherosclerosis.^22^ The present study showed that myeloid cell‐specific expression of *lncAPAT* promoted the expression of *MMP9* in aortic plaques and in monocytes isolated from mice. The expression of *COL1A1* was also decreased. TNFα‐induced upregulation of MMPs and downregulation of collagen further destabilised the plaque lesions.

In this study, *lncAPAT* overexpression in THP‐1 monocytes significantly increased the expression of the cytokine *ICAM‐1*. While it is well established that endothelial *ICAM‐1* binds monocyte lymphocyte function‐associated antigen‐1 to regulate adhesion and activation, it has also been reported that monocytes express *ICAM‐1* in an inducible manner and contribute to adhesion dynamics and inflammatory signalling. Linden et al. found that isolated human monocytes display very low *ICAM‐1* at baseline, but IFN‐γ treatment can double its expression, indicating that monocyte *ICAM‐1* may be dynamically regulated during the process of monocyte activation and transmigration.^23^ These results indicate that *lncAPAT* is crucial for the monocyte and macrophage inflammatory response, and that it promotes atherosclerosis progression.

We also observed that *lncAPAT* had a proinflammatory effect in HUVECs and HASMCs. Overexpression of *lncAPAT* increased the expression of proinflammatory cytokines, including *MCP‐1*, *IL‐1β*, *IL‐6*, and *TNFα*, in HUVECs, as well as *TNFα* in HASMCs. Macrophages play a critical role in atherosclerosis pathogenesis, driving plaque progression through several mechanisms, such as inflammation, lipid uptake (foam cell formation), and necrotic core development.^14^, ^15^ Importantly, *lncAPAT* exhibited the highest expression in macrophages compared with other vascular cell types, such as endothelial cells, smooth muscle cells, and aortic adventitial fibroblasts. Therefore, we prioritised investigating the function of *lncAPAT* in macrophages to elucidate its mechanistic role in atherosclerosis development and progression.

The findings showed that *lncAPAT* overexpression promoted the expression of inflammatory genes in peritoneal macrophages and PBMCs. In BMDMs, *lncAPAT* overexpression affected the expression of MMPs and collagen. These divergent transcriptional responses may be attributed to differences in the cellular origins and maturation states.^24^ Circulating monocytes, which originate from the bone marrow or splenic haematopoietic stem and progenitor cells, readily differentiate into proinflammatory M1 macrophages and are highly responsive to inflammatory stimuli.^25^ It is possible that systemic or local inflammatory responses may have been triggered by *lncAPAT* overexpression, leading to the upregulation of inflammatory cytokines. Peritoneal macrophages, conversely, are primarily tissue‐resident cells derived from embryonic progenitors. They exhibit high immune responsiveness and strong adaptation to their tissue microenvironment.^26^ In contrast, BMDMs differentiate in vitro under macrophage colony‐stimulating factor stimulation, which tends to skew them towards a repair‐associated or regulatory phenotype. This could limit their inflammatory response and account for the distinct transcriptional profile observed upon *lncAPAT* overexpression. These findings collectively suggest that macrophages from different anatomical regions and developmental contexts are functionally specialised. For instance, PBMCs and peritoneal macrophages are more inflammation‐prone, whereas BMDMs are more involved in matrix remodelling. Accordingly, *lncAPAT* may enhance pre‐existing, cell type‐specific gene programs rather than exerting uniform effects across all macrophage populations. Although some discrepancies were observed, the data collectively support that *lncAPAT* facilitates inflammatory responses, as evidenced by the consistently increased expression of *MCP‐1* across all three macrophage models. Given that *MCP‐1* was a key target in the present study, this finding provides strong support for the proinflammatory role of *lncAPAT*. The consistent upregulation of *MCP‐1* highlights its role as a robust downstream effector of *lncAPAT*.

Traditionally, atherosclerosis begins with lipoprotein accumulation in the subendothelium at sites of hemodynamic disturbance.^27^, ^28^ The present study found that *lncAPAT* overexpression promoted cholesterol accumulation, while *lncAPAT* knockdown inhibited cholesterol accumulation and increased the cholesterol efflux ratio in THP‐1‐derived macrophages. Consistently, *lncAPAT* increased cholesterol accumulation and decreased the cholesterol efflux ratio in monocytes isolated from *lncAPAT^flox/flox^;LyzM*‐Cre^+/−^ mice. These results indicate that *lncAPAT*‐induced cholesterol accumulation in macrophages may be an important factor contributing to the inflammatory response and plaque vulnerability.

RPL22 is an RNA‐binding protein component of the 60S ribosomal subunit that plays an indispensable role in global protein synthesis. Increasing evidence underscores the role of ribosomal proteins in regulating essential cellular processes. RPL22 can induce defects in ribosome synthesis, subsequently leading to distinct alternative splicing patterns that activate p53‐dependent transcriptional responses and arrest cell proliferation under ribosomal or nucleolar stress.^29^ A recent study showed that RPL22 accumulates within the nucleolus of senescent human mesenchymal progenitor cells and triggers a cascade of heterochromatin decompaction, playing a newly recognised role in the aging process.^30^ In studies of inflammation, a previous study observed a direct interaction between *MCP‐1* mRNA and RPL22 protein using crosslinking immunoprecipitation experiments. Moreover, the ultraviolet crosslinking assay clarified the essential role of the initial 20 bp segment within the 5′‐untranslated region of *MCP‐1* mRNA for RPL22 binding. Co‐immunoprecipitation with anti‐RPL22 antibody and liquid chromatography–mass spectrometry analysis confirmed the physical interaction between RPL22 and up‐frameshift protein 1 in LPS‐treated THP‐1‐derived macrophages and MCF7 cells.^17^ Up‐frameshift protein 1 plays a vital role in mRNA decay pathways, where it is ready to react in the event of an incorrect translation mechanism.^31^ The complex of RPL22 and up‐frameshift protein 1 has been shown to be responsible for *MCP‐1* mRNA degradation.^17^ Our RNA immunoprecipitation assays confirmed the direct interaction between RPL22 protein and *MCP‐1* mRNA in BMDMs, and we found that the mRNA and protein expression of *MCP‐1* was increased in macrophages after the inhibition of *RPL22* transcript activity by *lncAPAT*.

This study has some limitations. First, although our study provides initial evidence that *lncAPAT* contributes to coronary plaque destabilisation, the relatively limited sample size and single‐centre design render these findings exploratory. Large multicentre studies are warranted to validate and extend our observations. Second, we found that *lncAPAT* was more highly expressed in CD3^+^ T cells than in CD14⁺ monocytes and macrophages from the peripheral blood of control individuals. In the present study, we focused on macrophages because they are the predominant immune cell population within atherosclerotic plaques and play dominant roles at all stages of atherosclerosis, including lesion initiation, foam cell formation, necrotic core expansion, plaque rupture or erosion, lesion regression, and inflammation.^14^, ^15^ Nonetheless, lymphocytes, particularly T lymphocytes, also contribute to inflammation and plaque progression through cytokine production and interactions with macrophages.^32^ Future studies are necessary to delineate the functional effects of *lncAPAT* in T cells and other immune subsets. Third, we used the *LyzM*‐Cre system to generate a monocyte/macrophage‐*lncAPAT* knock‐in mouse model. Because *LyzM*‐Cre is commonly used to target myeloid cells, in addition to monocytes and macrophages, it also induces *lncAPAT* knock‐in in neutrophils owing to the expression of lysozyme in these cells. Therefore, the potential contribution of neutrophils to the observed phenotype cannot be excluded. Future studies using more specific Cre drivers or alternate genetic models that can differentiate between monocytes and neutrophils would provide a clearer understanding of the monocyte‐ and macrophage‐specific roles of *lncAPAT*. Fourth, the myeloid cell‐specific *lncAPAT* knock‐in mice model was generated via a CAG‐loxP‐Stop‐loxP cassette containing three tandem SV40 polyadenylation sequences (3 × SV40 pA), which may not completely abolish transcription in Cre‐negative controls and could allow low‐level basal *lncAPAT* expression. However, we did not detect differences between *lncAPAT^flox/flox^* and wild‐type mice at baseline in serum biochemical parameters, immune cell composition, or vascular phenotype, indicating that the potential low‐level basal *lncAPAT* expression in Cre‐negative controls could not remarkably affect experimental results in the present study. Fifth, our current study employed a constitutive *lncAPAT* knock‐in mouse model, which did not result in detectable baseline phenotypic alterations. For future investigations aiming to achieve precise spatiotemporal control of *lncAPAT* expression, conditional systems such as Cre‐dependent or inducible models could be considered. Finally, although we showed that *lncAPAT* interacted with *RPL22* to enhance *MCP‐1* transcription and promote inflammation in vitro, animal models with myeloid cell‐specific overexpression of *lncAPAT* and knock‐in of *RPL22* would be useful to verify the regulation of *RPL22* and *MCP‐1* by *lncAPAT* in vivo.

Cell‐free nucleic acids, such as circulating *lncRNAs*, have been proposed as potential biomarkers for the diagnosis and prognosis of cardiovascular disease.^33^ In the present study, *lncAPAT* was shown to be highly expressed in the peripheral blood of patients with CAD with mixed plaques compared with control individuals, suggesting that *lncAPAT* reflects underlying vascular inflammation and plaque activity. The enhanced expression of *lncAPAT* in the peripheral blood makes it an attractive candidate biomarker for clinical screening and cardiovascular disease monitoring. Furthermore, this study showed that *lncAPAT* contributes to atherosclerotic plaque instability by targeting the *RPL22*/*MCP‐1* axis, making it a potential therapeutic target for atherosclerotic plaque progression. For example, targeting *lncAPAT* with antisense oligonucleotides or RNA interference technologies could potentially attenuate vascular inflammation and plaque instability. However, clinical trials are required to verify its role in atherosclerosis.

## 5 CONCLUSIONS

**FIGURE 7:**
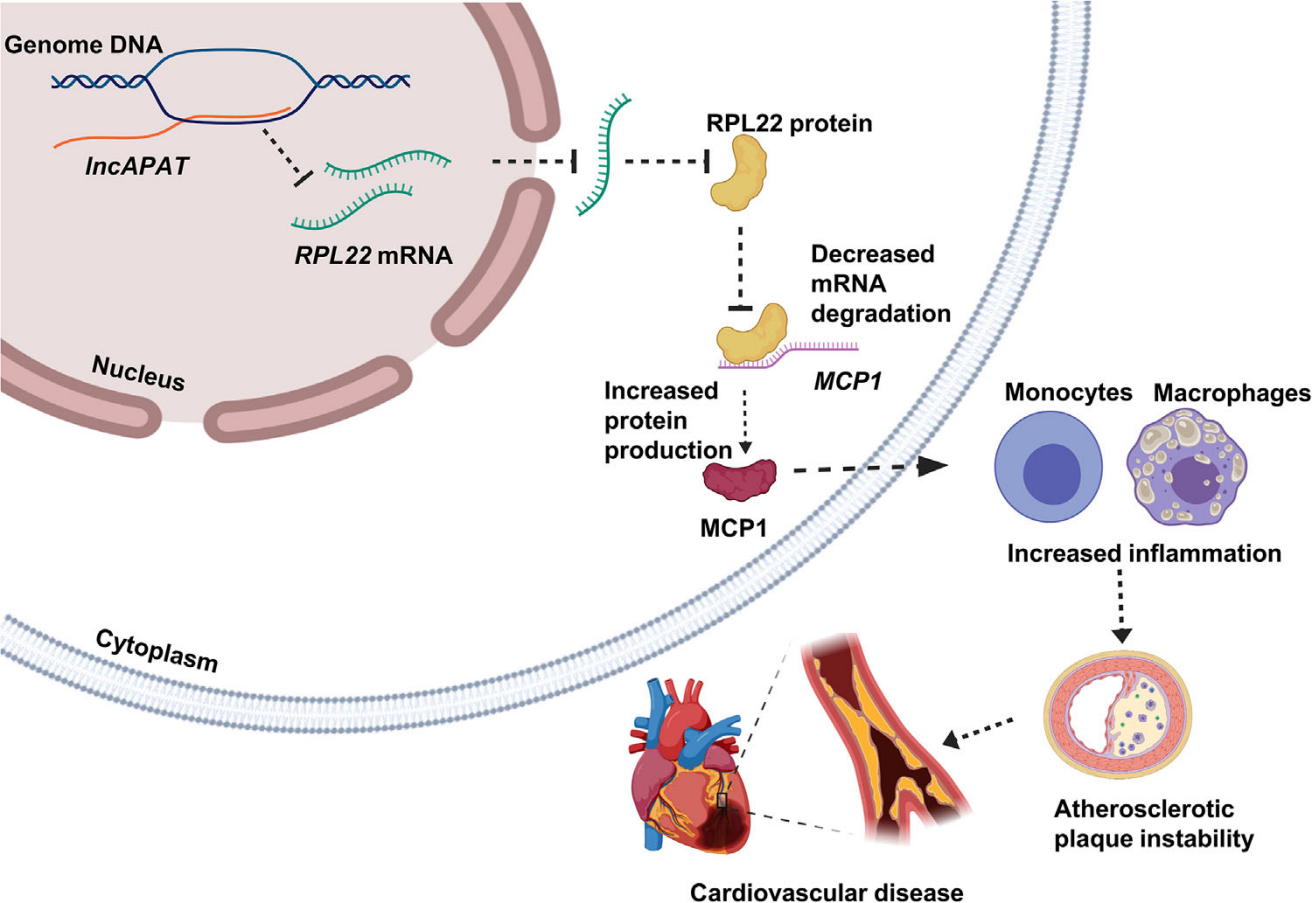
Schema depicting lncAPAT binding to the RPL22 promoter to repress RPL22 transcription, thereby enhancing MCP‐1 expression and promoting plaque instability.

In this study, we identified a new *lncRNA*, *lncAPAT*, which was highly expressed in the peripheral blood of patients with unstable coronary artery plaques. *LncAPAT* promoted the macrophage inflammatory response and atherosclerotic plaque progression by inhibiting the transcriptional activity of *RPL22*, indicating its potential as a therapeutic target for atherosclerosis (Figure 7). The identification of *lncAPAT* as a facilitator of macrophage inflammation expands our mechanistic insights into the role of *lncRNAs* in atherosclerotic plaque instability.

## AUTHOR CONTRIBUTIONS

Weili Zhang contributed to the study conception and design, and supervised the study. Rongxia Li, Qiyue Zhang, Yu Chen, Shuting Wang, Shuang Han, Adalaiti Kamili, Yixuan Zhong, and Shujun Yang performed the experiments and analysed the data. Rongxia Li and Qiyue Zhang wrote the manuscript. All the authors read and approved the final manuscript submitted.

## CONFLICT OF INTEREST STATEMENT

The authors declare no conflicts of interest.

## Supporting information



Supporting Information

## Data Availability

The data underlying this article will be shared on reasonable request to the corresponding author (Zhang W). All patients in this study provided written informed consent. The study protocols were conducted in accordance with the principles outlined in the Declaration of Helsinki and were approved by the Ethics Committee of Fuwai Hospital, China (Approval No.2021‐1445). All experiments involving animals were conducted according to the ethical policies and procedures approved by the ethics committee of FuWai Hospital, China (Approval No. FW‐2021‐0021).
