# Supplementary material for: Long non‐coding RNA lncAPAT promotes atherosclerotic plaque instability by targeting ribosomal protein L22
Source: Clin Transl Med. 2026 Jan 13;16(1):e70564. doi: 10.1002/ctm2.70564 (PMC12796838; doi:10.1002/ctm2.70564)

**Supplementary Materials**

**Full title:** Long noncoding RNA *lncAPAT* promotes atherosclerotic plaque instability by targeting ribosomal protein L22

**Short title:** *LncAPAT* promotes plaque instability

**Category:** Original article

**Authors and affiliations:** Rongxia Li^1†^ Ph.D., Qiyue Zhang^2†^ Ph.D., Yu Chen^1^ M.S., Shuting Wang^1^ Ph.D., Shuang Han^1^ M.S., Adalaiti Kamili^1^ M.S., Yixuan Zhong^1^ M.S., Shujun Yang^3*^ Ph.D., Weili Zhang^2,4,5*^ Ph.D.

^1^ State Key Laboratory of Cardiovascular Disease, FuWai Hospital, National Center for Cardiovascular Diseases, Chinese Academy of Medical Sciences and Peking Union Medical College, Beijing 100037, China.

^2^ National Clinical Research Center of Cardiovascular Diseases, FuWai Hospital, National Center for Cardiovascular Diseases, Chinese Academy of Medical Sciences and Peking Union Medical College, Beijing 100037, China.

^3^ Department of Cardiovascular Medicine, Xiangya Hospital, Central South University, Changsha, P.R. China; Center of Coronary Circulation, Xiangya Hospital, Central South University, Changsha, P.R. China; National Clinical Research Center for Geriatric Disorders, Xiangya Hospital, Central South University, Changsha 410008, P.R. China.

^4^ Central China Subcenter of National Center for Cardiovascular Diseases, Henan Cardiovascular Disease Center, Fuwai Central-China Cardiovascular Hospital, Central China Fuwai Hospital of Zhengzhou University, Zhengzhou 450000, China.

^5^ Institute of Cardiovascular Disease, Henan Academy of Innovations in Medical Science, Zhengzhou, Henan 451162, China.

† Rongxia Li and Qiyue Zhang contributed equally to this work.

**Correspondence to:**

Shujun Yang, Xiangya Hospital, Central South University, Hunan, China, Tel: +(86)17310608375, email: yangshujun0421@csu.edu.cn; or Weili Zhang, FuWai Hospital, National Center for Cardiovascular Diseases, Chinese Academy of Medical Sciences and Peking Union Medical College, Beijing, China, Tel: +(86)13910524025, e-mail: [zhangweili@fuwaihospital.org](mailto:zhangweili@fuwaihospital.org.com).

**1. Expanded Methods**

**1.1 Whole transcriptome sequencing and data analysis**

Total RNA was extracted using the Tempus™ Spin RNA Isolation Kit (ThermoFisher, USA) from the peripheral circulating blood of patients with coronary mixed plaques and control subjects without plaques. The procedure was as follows: blood samples in the Tempus™ Blood RNA Tube were poured into a clean 50-mL conical tube, and then 3 mL of phosphate-buffered saline (Gibco, USA) was added to the conical tube to bring the total volume to 12 mL. The tube was vortexed vigorously for 30 seconds to ensure proper mixing of the contents and centrifuged at 4°C at 3,000×g for 30 minutes. The supernatant was poured oﬀ carefully, and a total of 400 μL of RNA purification resuspension solution was added into the conical tube to resuspend the RNA pellet. Finally, the total RNA was purified with a purification filter and eluted with nucleic acid purification elution solution.

Strand-specific libraries were prepared using the TruSeq® Stranded Total RNA Sample Preparation kit (Illumina, USA) following the manufacturer’s instructions. To confirm the insert size and calculate the mole concentration, purified libraries were quantified by Qubit® 2.0 Fluorometer (Life Technologies, USA) and validated by Agilent 2100 bioanalyzer (Agilent Technologies, USA). RNA sequencing was conducted using the Illumina HiSeq X-ten (Illumina, USA). Sequencing raw reads were filtered by Seqtk before mapping to the human GRCh38 reference genome using Hisat2 (version 2.0.4). Differentially expressed long non-coding RNAs (lncRNAs) were identified using edgeR and selected with the filter criteria: fold change ≥ 2, false discovery rate ≤ 0.01, and Fragments Per Kilobase Million ≥ 1. The Ensembl database (https://asia.ensembl.org/index.html) was used for annotating the biotype of each transcript. The library construction and sequencing were performed at Shanghai Biotechnology Corporation.

**1.2 Phylogenetic construction**

The phylogenetic tree was used to analyze the homology of *lncAPAT* among different species. Through NCBI Blast analysis, a total of 20 homologous sequences of *lncAPAT* were identified*.* The *lncAPAT* gene sequences were aligned using the ClustalW method with default parameters, and a phylogenetic tree was constructed using the neighbor-joining method in MEGA11 with the following parameter settings: bootstrap value of 1000, p-distance model, and partial deletion for gaps/missing data^1^.

**1.3 Northern Blot Assay**

To confirm the transcript of *lncAPAT*, a northern blot assay was performed using the DIG Northern Starter Kit (Roche, Switzerland) according to the manufacturer’s protocol. Briefly, total RNA (20 µg) from THP-1 cells was separated by 1% formaldehyde-agarose gel and transferred to a nylon membrane by the capillary siphon method. The nylon membrane was prehybridized with DIG Easy Hyb for 30 min. In the meantime, the digoxigenin-labeled RNA probe was denatured by boiling for 5 min and rapidly cooled in ice. Next, the nylon membrane was hybridized with denatured digoxigenin-labeled RNA probe (100 ng/mL) and hybridization mixture for 6 h at 68℃ with gentle agitation. The digoxigenin-labeled RNA probe was obtained from *in vitro* transcription with a DNA template. The DNA template was prepared from the total RNA of THP-1 monocytes using RT-PCR and PCR. The promoter of T7 RNA polymerase was used for the PCR reaction, and the sequence of designed primers were as follows: primer F1: 5’-GCCATCCTAGACCACTGA-3’, primer R1: 5’-TAATACGACTCACTATAGGGGTTACTAGCACTGTCCTCAT-3’; primer F2: 5’- GCTGTCTGAATGATCTGAG-3’, primer R2: 5’- TAATACGACTCACTATAGGGCTGCTACCTTGTGGAGTT-3’; The hybridized probes were visualized with the FluorChem R, M and E Systems (ProteinSimple, USA).

**1.4 Fluorescent *in situ* Hybridization**

For subcellular localization of *lncAPAT* in macrophages derived from THP-1 cells, RNA FISH was conducted using the Fluorescent In Situ Hybridization Kit (RiboBio, China) in accordance with the manufacturer’s directions. Briefly, macrophages at 50% confluency were fixed with 4% paraformaldehyde for 10 min at room temperature. After being washed three times with PBS buffer, the macrophages were permeabilized with 0.5% Triton X-100 (Solarbio, China) in PBS buffer for 5 min at 4℃. Cy3-conjugated *lncAPAT* FISH probe mix, *h-18S* FISH probe mix, and *h-U6* FISH probe mix were respectively mixed with probe diluent at a concentration of 0.5 μM. Followed by the hybridization of macrophages and probes at 37 °C for 18 hours. The nuclei were stained with 4',6-diamidino-2-phenylindole (DAPI) (ZSGB-BIO, Beijing, China). *h-18S* and *h-U6* were used as the cytoplasmic and nuclear positive controls, respectively. All images were captured using a confocal laser scanning microscope (SP8, Germany).

**1.5** **Cytoplasmic and Nuclear RNA Purification**

Cytoplasmic and nuclear RNA of macrophages derived from THP-1 cells were separated using a Cytoplasmic & Nuclear RNA Purification Kit (NORGEN, Thorold, Canada). Briefly, a total of 1×10^6^ cells were lysed using ice-cold lysis buffer for 5 minutes with gently tapping and swirling the culture dish on ice. Then, the lysate was collected and centrifuged at 14000 g for 10 minutes to separate the supernatant containing cytoplasmic RNA and the precipitate containing nuclear RNA. Cytoplasmic and nuclear RNA were extracted and purified with a spin column. Equal amounts of cytoplasmic and nuclear RNA were used for reverse transcription and qRT-PCR detection. The expression of *lncAPAT* in the cytoplasm and nucleus was normalized to ribosomal protein *S14* and *U2* small nuclear ribonucleoprotein, respectively. *GAPDH* was used for a positive control. The percentage of *lncAPAT* in the nucleus was calculated with the following formula:

$$\mathrm{Nucleus} \% =\frac{2-CT(Nucleus)}{2-CT(Nucleus)+ 2-CT(Cytoplasmic)}$$

The percentage of *lncAPAT* in the cytoplasm was 1- Nucleus %. The primers were listed in Table S3.

**1.6 Chromatin Isolation by RNA Purification-Sequencing**

To identify specific regions of genomic DNA interacting with *lncAPAT*, chromatin isolation by RNA purification (ChIRP) assay was conducted using an EZ-Magna ChIRP RNA Interactome Kit (Millipore, MA, USA). Briefly, anti-sense biotinylated DNA tiling probes corresponding to the full length of *lncAPAT* were designed and synthesized by RiboBio (Guangzhou, China). The CHIRP_Probe sequences were as following: ChIRP Probe_1: ATCTCTGGATGTATGCTGCT-/3bio/; ChIRP Probe_2: ACTTGATAGGTGGTGAGTCC-/3bio/; ChIRP Probe_3: TTGTGGAGTTCTGTTTCTTC-/3bio/; ChIRP Probe_4: CCCATCTACATTCCCATAAC-/3bio/; ChIRP Probe_5: CCGAGACAGCTACTGATAAT-/3bio/.

A total of 2×10^7^ THP-1-derived macrophages or bone marrow-derived macrophages (BMDMs) from *lncAPAT^flox/flox^;LyzM*-Cre^+/-^ mice were cross-linked with 1% glutaraldehyde (Sigma-Aldrich, MO, USA) at room temperature (18-25ºC) for 10 minutes on an end-to-end shaker. Followed by cell lysis with 1 ml of completed lysis buffer per 100 mg cell pellet. Proceed immediately to sonicate for chromatin shearing into a smear of DNA in the range of 100 bp to 500 bp with the Covaris S220 Focused Ultrasonicator. At this point, 10 µL of sonicated cell lysate was taken as “RNA input”. Next, sonicated cell lysate was hybridized with *lncAPAT* CHIRP_probes at 37 °C for 4 hours with end-to-end mixing. After 4 hours hybridization reaction, 100 µL of streptavidin magnetic beads were added into the tube at 37 °C for an additional 30 minutes with constant mixing to capture the chromatin bound with *lncAPAT*. RNA from the enriched product was isolated using the QIAGEN miRNeasy^®^ Mini Kit (QIAGEN, Dusseldorf, Germany) and analyzed using an iTaq Universal One-Step Kit (Bio-Rad, Hercules, California, USA).

DNA from the enriched product was sequenced on the NovaSeq 6000 platform (Illumina, USA). MACS2 was used for peak calling of the ChIRP-seq data with a set of parameters. The input data was in BAM format, and signal normalization was applied using the signal per million reads option (SPMR) to facilitate accurate comparison and visualization. A p-value threshold of 0.01 was used to identify statistically significant peaks. The genome size was set to human (for macrophages differentiated from THP-1 cells) or mouse (for BMDM cells from mice). The default model-building step was skipped to accommodate the unique characteristics of ChIRP-seq data, which often do not conform to standard fragment distribution assumptions. Instead, a fixed fragment length of 200 bp was specified with no shift applied. All duplicate reads were retained to preserve biologically relevant redundancy typical in ChIRP-seq experiments.

To validate whether the *lncAPAT* is truly binding to the identified genomic regions. RNA retrieval, which refers to the recovery of *lncAPAT* after it has been enriched and pulled down with biotinylated antisense probes, was quantified with qRT-PCR method. RNA retrieval (%) was calculated with the formula below:

$$RNA Retrieval \left( \% \right)=2^{-\left( C_{T}^{Pulldown}-\left( C_{T}^{Input}-\log_{2} 10 \right) \right)}\times100$$

To assess the DNA locus truly interacting with *lncAPAT* was pulled down, primers flanking these peak regions were designed, and the qRT-PCR was conducted after CHIRP-seq. Primers were shown in Table S3. CHIRP assay was conducted twice for each type of cells (macrophages and BMDMs) for a second validation.

**1.7 Genotyping Methods of Genetic Animal Model**

Myeloid cell-specific *lncAPAT* knock-in mice were generated by Cyagen Biosciences (Suzhou, China). The *lncAPAT^flox/flox^* mice (control mice) and *lncAPAT^flox/flox^;LyzM*-Cre^+/-^ mice (experimental mice) were genotyped by PCR with primer 1, primer 2, and primer 3. The sequences of designed primers were as follows: primer F1: 5’- AGATCTGCAAGCTAATTCCTGC-3’, primer R1: 5’- ACTTAGCTCAGATCATTCAGACAG-3’, with a product of 600 bp; primer F2: 5’- AAGCACGTTTCCGACTTGAGTTG-3’, primer R2: 5’- GGGTGAGCATGTCTTTAATCTACC-3’, with a product of 160 bp; primer F3: 5’-CCCAGAAATGCCAGATTACG-3’, primer R3: 5’-CTTGGGCTGCCAGAATTTCTC-3’, with a product of 700 bp. The genomic DNA from the tail of *lncAPAT^flox/flox^* mice produced one band of 160 bp, and that from *lncAPAT^flox/flox^;LyzM*-Cre^+/-^ mice produced two bands of 160 bp and 700 bp.

**1.8 Atherosclerotic Lesion Analysis**

The atherosclerotic lesions in the aorta were assessed by en face analysis and cross-sectional measurement. For en face analysis, mouse aortas were placed in cold normal saline, cleaned of fatty tissue, and incised longitudinally at the thoracic aorta and aortic arch. The aortas were stained with 0.3% Oil red O (Sigma-Aldrich, USA) for 30 min prior to visualization. Images were captured with a stereomicroscope-dedicated camera (Zeiss, Jena, Germany) and analyzed with Image-Pro Plus 6.0. The lesion area was shown as a percentage of the total surface area.

For the analysis of plaque morphology, fresh isolated heart and aortic plaque tissues were frozen in Tissue-Tek O.C.T. Compound (Sakura, USA), and 8 μm sections were continuously cut. Aortic root sections were collected when all three valves were clearly visible. Thoracic aortic sections were collected from the most stenotic part of the plaque. Three sections of aortic root and thoracic aortic, 50 μm apart per mouse were grouped for staining with hematoxylin-eosin staining using varistain^TM^ gemini (Thermo, Waltham, MA, USA) for quantifying the lesion area and necrotic core area, Oil Red O for quantifying the lipid content in plaque, Picrosirius Red (Leagene, Beijing, China) for the ratio of type1/3 collagen, Masson’s trichrome (Leagene Biotechnology, Beijing, China)for the total collagen and Van Gieson (VG) (Sigma-Aldrich, MO, USA) for the degradation of elastic fiber. These quantifications were performed in a blinded manner.

**1.9 Immunofluorescence Staining**

OCT-embedded frozen sections of mouse thoracic aortas were used for immunofluorescence staining. Briefly, frozen sections (8 μm thick) were incubated with a rabbit anti-mouse CD68 antibody (Abcam, Cambridge, England), mouse matrix metallopeptidase (MMP) 2 (Abcam, Cambridge, England), mouse MMP9 (Abcam, Cambridge, England), mouse α-smooth muscle actin (α-SMA) (Abcam, Cambridge, England), mouse CD3 antibody (Abcam, England), mouse Ly6G antibody (Abcam, England) and mouse Ribosomal protein L22 (RPL22) (Santa Cruz, California, USA), respectively. Followed by incubation with an Alexa Fluor® 488-labelled goat anti-rabbit IgG secondary antibody (green) (ZSGB-BIO, China) for CD68, MMP2, MMP9, αSMA, CD3, Ly6G, and RPL22; or Alexa Fluor® 594-labelled goat anti-rabbit IgG secondary antibody (red) (ZSGB-BIO, China) for CD68 co-staining with RPL22. Nuclei were counterstained with 4',6-diamidino-2-phenylindole (DAPI). Images were captured with a confocal laser scanning microscope (SP8, Leica, Germany).

**1.10 Lipid Profile Analysis**

Serum samples were separated from mouse orbital blood by centrifugation at 8000 rpm for 5 min at 4℃ for the detection of lipid profiles. Total cholesterol and triglycerides in serum were measured using an automatic dry biochemical analyzer (NX700IV, Tokyo, Japan). High-density lipoprotein cholesterol (HDL-C) and low-density lipoprotein cholesterol (LDL-C) were measured by the double reagent direct method using the high-density lipoprotein cholesterol assay kit and Low-density lipoprotein cholesterol assay kit (Nanjing Jiancheng Bioengineering Institute, China), respectively, according to the manufacturer’s directions. Briefly, A total of 2.5 μL serum sample was mixed with 180 μL R1, followed by incubating at 37 °C for 5 min. The absorbance value (A1) was determined at 546 nm by using the LB960 microplate reader (Berthold, Bad Wildbad, Germany). Next, the mixture was added with 50 uL R2 and incubated at 37 °C for 5 min. The absorbance value (A2) was determined again at 546 nm by using the LB960 microplate reader. Distilled water and the standard were used as blank control and positive control, respectively. The concentration of HDL-C or LDL-C was calculated with the following formula:

$$(mmol/L) =\frac{\left( Sample A2-Sample A1 \right)-\left( Control A2-Control A1 \right)}{\left( Standard A2-Standard A1 \right)-(Control A2-Control)}\times standard(mmol/L)$$

**1.11 Isolation and Culture of Mouse** **Peritoneal Macrophages**

The limbs of anesthetized mice were attached to the foam board, and the abdomen was sterilized with 70% ethanol, followed by injection of 5 mL serum-free RPMI-1640 medium into the peritoneal cavity using a 5 mL sterile syringe with a 25-gauge needle. The mouse abdomen was gently massaged for 5 minutes. Then, a small incision in the abdomen was made with a sterile scalpel, and the medium was collected into a 15 mL tube as much as possible. The medium containing peritoneal macrophages was centrifuged for 5 min at 310 × g at 4 °C to pellet cells. The supernatant was discarded, and the peritoneal macrophages were resuspended and washed twice with PBS. Next, the peritoneal macrophages were cultured in serum-free RPMI-1640 medium with a density of 1×10^6^ cells/mL for 2 h and finally collected into TRIzol reagent for total RNA extraction.

**1.12 Isolation and Culture of Mouse Peripheral Blood Mononuclear Cells**

A total of 600 μL of blood sample of mice was obtained via cardiac puncture into a 5 mL heparin tube. The blood sample was centrifuged for 10 minutes at 2000 rpm at room temperature. The cells in the middle layer were collected into a 15 mL centrifuge tube, and the same volume of PBS was added. A total of 2 mL Histopaque-1077 (Sigma-Aldrich, USA) was added to another 15 mL tube. The sample was added into the 15 mL tube with Histopaque-1077 along the wall gently, followed by a centrifuge for 35 minutes at 1500 rpm. The sample in the middle layer was collected and diluted in 10 mL PBS. Then, the sample was centrifuged for 10 minutes at 1300 rpm, followed by washing twice with PBS. The cells were resuspended in serum-free RPMI-1640 medium, seeded in a 12-well plate at a density of 1×10^6^ cells/mL, cultured for 2 hours, and finally collected into TRIzol regent for total RNA extraction.

**1.13 Isolation and Culture of Mouse Bone Marrow-derived Macrophages**

The hind legs of mice were sterilized with 70% ethanol by soaking all the fur. The femur and tibia were harvested by cutting the hind legs through the pelvis close to the hip joint. The muscle tissues of bones were removed using a scalpel and dissecting scissors. The bones of each mouse were then collected into a 10 cm dish with precooled PBS buffer and washed briefly with 70% ethanol to ensure sterility. Next, epiphyses at the ends of each bone were removed using sterile scissors and forceps, and total bone marrow was isolated by flushing each bone with precooled PBS buffer 15 times until the bone became transparent. The bone marrow washed out by PBS was centrifuged for 10 min at 1500 rpm, and the collected cells were resuspended in 1 mL red blood cell lysing buffer hybrid-max^TM^ (Sigma-Aldrich, USA). After 5 minutes, a total of 10 mL PBS was added, and the cells were centrifuged again for 10 min at 1500 rpm. The bone marrow-derived macrophages were resuspended in 10 mL serum-free RPMI-1640 medium and seeded in a 10 cm cell culture dish. After 2 hours, the cultural supernatant was collected in a 50 ml centrifuge tube and centrifuged for 10 min at 4 °C at 1500 rpm. Finally, bone marrow-derived macrophages were resuspended in DMEM medium supplied with 10% FBS, penicillin (100 U/mL), streptomycin (100 μg/mL), and 50 ng/mL macrophage colony-stimulating factor (M-CSF) (PeproTech, Rocky Hill, USA) and seeded in a 6-well plate with a density of 1×10^6^ cells/mL. A complete change of fresh culture medium was supplied on day 3, and bone marrow-derived macrophages were collected in TRIzol reagent on day 7 for subsequent experiments.

**1.14 Isolation and Culture of** **Bone Marrow Neutrophils**

Morphologically mature neutrophils were purified from mouse bone marrow as described previously^2^. In brief, skin and musculature were removed to expose hind limbs, and femurs and tibias were excised, rinsed in 70% ethanol, and washed three times in ice-cold PBS. Bone ends were cut off, and marrow was flushed through a 100-µm cell strainer into RPMI-1640 containing 10% FBS and 2 mM EDTA, using a 25-gauge needle. Residual marrow fragments were gently dissociated by triturating bone chips through the strainer. Cells were pelleted at 427 × g for 7 min at 4 °C and then layered onto a discontinuous Histopaque gradient (1.119 g/ml under 1.077 g/ml) at room temperature and centrifuged for 30 min at 872 × g without brake. Neutrophils were harvested from the 1.077/1.119 interface, washed twice in RPMI-1640/10% FBS/1% penicillin/streptomycin, and pelleted at 1,400 rpm for 7 min at 4 °C. Finally, the mouse bone marrow neutrophils were collected into TRIzol regent for total RNA extraction.

**1.15 Cell Culture**

The human acute monocytic leukemia cell line, THP-1 cells (China Infrastructure of Cell Line Resources, China), were cultured in RPMI-1640 Medium (HyClone, USA) with 10% fetal bovine serum (FBS) (Gibco, USA). THP-1 cells were differentiated to M0 macrophages with stimulation of 100 ng/mL phorbol-12-myristate-13-acetate (Sigma-Aldrich, USA) for 48 hours. M1 or M2 macrophages were respectively induced by 1 ng/mL lipopolysaccharide (LPS) (Sigma-Aldrich, USA) and 20 ng/mL interferon (IFN)-γ (PeproTech, USA) for 24 hours, or by 20 ng/mL interleukin (IL) -4 (PeproTech, Rocky Hill, NJ, USA) for 24 hours.

The primary human umbilical vein endothelial cells (HUVECs) (ScienCell, USA) were cultured in the endothelial cell medium (ECM) (ScienCell, USA), adding 5% FBS, penicillin (100 U/mL), streptomycin (100 μg/mL), and 50 μg/mL endothelial cell growth supplement (ECGS). Human aortic endothelial cells (ScienCell, USA) were cultured in EBM-2 bullet kit media (Lonza, Basel, Switzerland) with 2% FBS, 0.04% hydrocortisone, 0.4% human fibroblast growth factor-basic, 0.1% vascular endothelial growth factor, 0.1% R3-insulin-like growth factor-1, 0.1% ascorbic acid, 0.1% human epidermal growth factor, 0.1% gentamicin, amphotericin-b and 0.1% heparin. Both human aortic smooth muscle cells (HASMCs) (ScienCell, USA) and human umbilical artery smooth muscle cells (ScienCell, USA) were cultured in smooth muscle cell medium (ScienCell, USA), supplemented with 2% FBS and 1% smooth muscle cell growth supplement (SMCGS). Human aortic adventitial fibroblast cells (ScienCell, USA) were cultured in fibroblast medium (ScienCell, USA), supplemented with 2% FBS and 1% fibroblast growth supplement (FGS). These cells were maintained at 37℃ in a humidified atmosphere containing 5% CO_2_.

**1.16 Small RNA Transfection and Lentiviral Infection**

LncRNA smart silencer (RiboBio, China) was used for *lncAPAT* knockdown. *LncAPAT* smart silencer is a mixture of three small interference RNAs (siRNAs) and three antisense oligonucleotides (ASOs), respectively targeting different sequences in the cytoplasm and nucleus. The target sequences of siRNAs are as follows: 5’-CCAGCTTGCATCAGAAGAA-3’, 5’-TGGACTCACCACCTATCAA-3’, 5’-GCAGCATACATCCAGAGAT-3’. The target sequences of ASOs are as follows: 5’-CAGAAGAACTGTCCAGAAAA-3’, 5’-CCAGAAAACTCACAGAATCA-3’, 5’-TAAGAAATAATGCAGCGCAG-3’. The sequence of the negative control does not contain domains homologous to humans and rats. The lncRNA smart silencer was transfected with Lipofectamine RNAiMAX reagent (Invitrogen, Carlsbad, CA, USA) at a concentration of 10 pM in THP-1 cells, macrophages derived from THP-1 cells, HUVECs, HASMCs, and BMDMs of mice according to the manufacturer’s instructions.

For the knockdown of RPL22 in THP-1-derived macrophages and BMDMs, a total of 60 nM RPL22 siRNA (GenePharma, Suzhou, China) was transfected using Lipofectamine 3000 (Invitrogen, Carlsbad, CA, USA) for 48 h. The target sequences of siRNAs are as follows: 5’-GCAAAGAGAGUUACGAGCUTT-3’, 5’-AGCUCGUAACUCUCUUUGCTT-3’. To establish a stable cell line of *lncAPAT*, THP-1 cells were infected with *lncAPAT* recombinant lentiviruses or control vectors (GeneChem, Shanghai, Company). Four days later, THP-1 cell lines stably expressing GFP or the GFP-tagged *lncAPAT* were selected by the FACS Aria 2 Flow Cytometry/Cell Sorting System (BD Biosciences, San Jose, CA, USA). To establish the stable cell lines of *lncAPAT* in HUVECs and HASMCs were used the same process was used as described above.

**1.17 Cell Adhesion Assay**

Cell adhesion assay was conducted to assess the effect of *lncAPAT* on THP-1 cells adhesive capacity to the endothelial cell monolayer. The human umbilical vein endothelial cells were seeded onto 24-well plates in ECM complete medium and grown to 90% confluency. The THP-1 cells were cultured in RPMI-1640 medium with 10% FBS, then harvested and washed twice with PBS. The cells were resuspended in serum-free RPMI-1640 medium with a density of 1×10^6^ cells/mL. One milliliter of the THP-1 cell suspension was labeled with 5 μL CellTracker^TM^ CM-Dil (Invitrogen, Carlsbad, CA, USA) for 30 minutes at 37 ℃. After being washed twice with PBS, the labeled THP-1 cells were resuspended again in the RPMI-1640 medium with 1×10^6^ cells/mL, added to each well (200 μL/well), and kept in an incubator with 5% CO_2_ at 37 ℃ for 30 minutes. The non-adherent THP-1 cells were removed by washing with PBS, and the adherent cells were counted in 5 different fields.

**1.18 Cholesterol Cellular Accumulation**

The effects of *lncAPAT* on the cholesterol accumulation of macrophages were assessed by Oil red O staining. Briefly, THP-1 monocytes were seeded into 12-well plates containing a coverslip and were differentiated to M0 macrophages as described above. After knockdown or overexpression of *lncAPAT*, macrophages were cultured in RPMI-1640 medium supplemented with 1% bovine serum albumin (BSA; Cat# A7030, Sigma-Aldrich, USA) at 37 °C for 12 h (16 h for mouse-derived macrophages) in the presence or absence of 50 µg/mL oxidized low-density lipoprotein (ox-LDL; Cat. No. YB-002, Yiyuan Biotech, China). After washing three times with PBS, cells were stained with 0.3% Oil red O for 3 min and counterstained with hematoxylin (Leagene, China) for 10 s at room temperature. The lipid accumulation status of cells was examined by light microscopic examination (DM6000B, Leica, Germany). The ratio of lipid deposit was calculated as the percentage of stained area to total cell area.

**1.19** **Cholesterol Efflux Assay**

The effect of *lncAPAT* on the cholesterol efflux ability of macrophages was assessed by fluorescence spectrometry with NBD cholesterol (22-(N-(7-Nitrobenz-2-Oxa-1,3-Diazol-4-yl) Amino)-23,24-Bisnor-5-Cholen-3β-Ol) (Thermo Fisher Scientific Inc., Waltham, MA, USA) analog (Invitrogen, N1148, USA). Briefly, macrophages were cultured at a density of 2 × 10⁶ cells/mL and incubated in RPMI 1640 medium supplemented with 0.2% bovine serum albumin (BSA) for 12 hours to equilibrate intracellular free cholesterol levels. Following equilibration, the cells were loaded with NBD-cholesterol (10 µg/mL) for 6 hours to facilitate cholesterol labeling. The cells were incubated in serum-free RPMI 1640 medium containing 50 µg/mL high-density lipoprotein (HDL) for 4 hours to induce cholesterol efflux. After the efflux incubation, the supernatant was carefully collected, and the cells were washed with phosphate-buffered saline (PBS) to eliminate residual medium. Cells were then lysed using 0.1% Triton X-100. Both the collected medium and the cell lysates were centrifuged separately at 2,000 rpm for 10 minutes to remove debris. Fluorescence intensity was measured using an Infinite M200 Pro Multimode Microplate Reader (Tecan Trading, China) with excitation at 469 nm and emission at 537 nm. The cholesterol efflux rate was calculated with the formula below:

$$\left( cholesterol efflux \% \right)=$$

$$\frac{\left( Fluorescence intensity of superment \right)}{\left( Fluorescence intensity of superment \right)+\left( Fluorescence intensity of lysed cells \right)}\times100\%$$

**1.20 Cell proliferation and migration assay**

To assess the effect of *lncAPAT* on HUVECs and HASMCs functions, cell proliferation and migration were conducted in Lv-*lncAPAT*-HUVECs, Lv-*lncAPAT*-HASMCs, Si-*lncAPAT*-HUVECs, Si-*lncAPAT*-HASMCs, and negative controls relatively. HUVECs were seeded in 96-well plates (Corning, China) at 5 × 10^3^ cells/well in complete ECM medium, whereas HASMCs were seeded at the same density in DMEM medium with 10 % FBS. Plates were placed in an IncuCyte® S3 Live-Cell analysis system (Sartorius AG, Germany). Images were captured every 2 h for up to 48h. Data were analyzed using the IncuCyte S3® system. In the cell proliferation assessment, dynamic live cell number changes were monitored, and cellular confluence (%) was calculated. The scratch test was used to assess the cell migration ability. Post-scratch cell confluence was calculated automatically by the IncuCyte S3® system, and > 5 different areas were analyzed. All experiments were performed using 96-well plates and repeated three times independently.

**1.21 RNA immunoprecipitation assay**

To analyze the interaction of protein RPL22 and *MCP-1* RNA in mouse bone marrow-derived macrophages, RNA immunoprecipitation was performed using the PureBinding RNA immunoprecipitation kit (Geneseed, China) according to the manufacturer’s instructions. The cells (1 × 10^7^ mouse bone marrow-derived macrophages) were homogenized in 1 mL immunoprecipitation lysis buffer with RNasin and protease inhibitor, then incubated on ice for 10 min to prepare the RIP lysate. Protein A/G Magnetic Beads were washed with Buffer A and then incubated with 5 µg ribosomal proteins RPL22 mouse monoclonal antibody (Santa Cruz, California, USA) or IgG as a negative control at 4°C for 30 min. Then the bead-antibody complexes were incubated with cell lysis buffer to capture the RNA-protein complex at 4°C overnight. After incubation, the samples were washed with Buffer B to eliminate non-specifically bound proteins and RNA. Then RNA was extracted from the RNA immunoprecipitation samples using RC Columns in the kit following the manufacturer’s guidelines. Reverse transcription of the extracted RNA into complementary DNA (cDNA) was performed using PrimeScript Reverse Transcriptase assay (Takara, China), adhering to the manufacturer’s protocol. The relative enrichment of mouse *MCP-1* mRNA was quantified by qRT-PCR using SYBR Green qPCR mix (YEASEN, China) on the ABI 7500 System (Applied Biosystems, Foster City, USA) and was calculated with the formula 2^–ΔΔCT^ [ΔΔCT= (Ct IP-CtInput)-(CtIgG-CtInput)]. The primers applied for qRT-PCR analysis were listed in Table S3. The RNA immunoprecipitation assay was performed independently in three biological replicates.

**1.22 RNA Extraction and Gene Expression Analysis**

Total RNA was extracted using TRIzol reagent (Invitrogen, USA), and cDNA was synthesized from 700 ng total RNA using PrimeScript Reverse Transcriptase assay (Takara, Dalian, China) according to the manufacturer’s instructions. The mRNA expression levels were analyzed by the qRT-PCR method using SYBR Green qPCR mix (YEASEN, China) on the ABI 7500 System (Applied Biosystems, USA). mRNA expression levels were normalized to *GAPDH* as endogenous control, and fold changes of relative gene expressions were calculated with the formula 2^–ΔΔCT^ (ΔCT=CT^target gene^-CT*^GAPDH^*). The relative *lncRNAs* expressions were calculated using the formula 2-^ΔCt^ (ΔCT=CT*^lncRNA^*-CT*^GAPDH^*). The primers applied for qRT-PCR analysis were listed in Table S3.

**1.23 Western Blot Assay**

Cells were lysed using RIPA buffer (Beyotime Biotechnology, China) with protease inhibitor (Roche, Mannheim, Germany) for proteins isolation. Protein extracts were subjected to NuPAGE 4-12% Bis-Tris Gel (Invitrogen, USA). Separated proteins were transferred to PVDF membranes using the iBlot 2 Gel Transfer Device (ThermoFisher Scientific, IB21001). The membrane was blocked with 5% nonfat milk for 1 hour at room temperature, followed by immunoblot analyses using antibodies against either RPL22 (Santa Cruz, USA), CCL2 (Abcam, England), and GAPDH (Cell Signaling Technology, USA). Quantification of protein bands was performed using a luminescent image analyzer (Tanon, China).

**References**

- - - 1. Tamura K, Stecher G, Kumar S. MEGA11: Molecular Evolutionary Genetics Analysis Version 11. Mol Biol Evol. 2021;38(7):3022-3027.
      2. Swamydas M, Luo Y, Dorf ME, et al. Isolation of Mouse Neutrophils. *Curr Protoc Immunol*. 2015;110:3.20.1-3.20.15.

**2 Supplementary Table and Figures**

This part includes 4 Tables and 19 Figures.

**Table S1.** Clinical characteristics of control subjects and coronary artery disease patients with mixed plaques on coronary computed tomography angiography.

**Table S2.** Clinical characteristics of control subjects, coronary artery disease patients with mixed plaques, and acute ST-elevation myocardial infarction patients undergoing optical coherence tomography examination.

**Table S3.** The primers for quantitative real-time polymerase chain reaction analysis.

**Table S4.** Potential targets of *lncAPAT* enriched by chromatin isolation by RNA purification assay (ChIRP).

**Figure S1.** The representative image of coronary artery disease patients with mixed plaque on coronary computed tomography angiography.

**Figure S2.** The validation of candidate transcripts by the qRT-PCR method in the peripheral blood of coronary artery disease (CAD) patients (n=5) who had coronary mixed plaques with coronary computer tomography angiography, compared with control subjects (n=10).

**Figure S3.** Agarose gel electrophoresis of RT-PCR products for *lncAPAT* and *GAPDH* in the peripheral blood of coronary artery disease patients (n=5) who had coronary mixed plaques with coronary computer tomography angiography and control subjects (n=10).

**Figure S4.** The expression of *lncAPAT* in monocytes (CD14+), B cells (CD19+), and T cells (CD3+) isolated by magnetic bead sorting from the peripheral blood of control subjects (n=4).

**Figure S5.** The phylogenetic tree of *lncAPAT*.

**Figure S6.** Establishment and validation of myeloid cell-specific *lncAPAT* knock-in mice.

**Figure S7.** The effect of *lncAPAT* on baseline phenotypes of wild-type, *lncAPAT^flox/flox,^* and *lncAPAT^flox/flox^;LyzM*-Cre^+/-^ mice before experimental intervention.

**Figure S8.** The effect of *lncAPAT* on serum biochemical markers and body weight of the *lncAPAT^flox/flox^* mice and *lncAPAT^flox/flox^;LyzM*-Cre^+/-^ mice.

**Figure S9.** The effect of *lncAPAT* on collagen content, lipid deposition, cap thickness, and elastic degradation grade in the thoracic aorta of the *lncAPAT^flox/flox^* mice and *lncAPAT^flox/flox^;LyzM*-Cre^+/-^ mice.

**Figure S10.** The effect of *lncAPAT* on atherosclerotic lesion areas of the aorta root of the *lncAPAT^flox/flox^* mice and *lncAPAT^flox/flox^*;*LyzM*-Cre^+/-^ mice.

**Figure S11.** The effect of *lncAPAT* on atherosclerotic lesion areas of the thoracic aorta in female *lncAPAT^flox/flox^* mice and *lncAPAT^flox/flox^;LyzM*-Cre^+/-^ mice.

**Figure S12.** Effects of *lncAPAT* on the function of human umbilical vein endothelial cells.

**Figure S13.** Effects of *lncAPAT* on the function of human aortic smooth muscle cells.

**Figure S14.** Agarose gel electrophoresis of RT-PCR products from the ChIRP assay.

**Figure S15.** The mRNA expression of potential targets of *lncAPAT* in the peripheral blood of

coronary artery disease (CAD) patients and acute ST-elevation myocardial infarction (STEMI) patients.

**Figure S16.** Agarose gel electrophoresis of RT-PCR products for *MCP-1* from the RIP assay in bone marrow-derived macrophages of the *lncAPAT^flox/flox^;LyzM*-Cre^+/-^ mice (n=3 in each group).

**Figure S17.** The effect of *lncAPAT* on the expression of RPL22 and MCP-1 in macrophages of thoracic aorta plaque in female *lncAPAT^flox/flox^* mice and *lncAPAT^flox/flox^;LyzM*-Cre^+/-^ mice.

**Figure S18.** The effect of *lncAPAT* –*RPL22* axis on the mRNA expression of *IL-6*, *IL-1β*, *TNFα*, *MMP-2*, and *MMP-9* in bone marrow-derived macrophages of *lncAPAT^flox/flox^;LyzM*-Cre^+/-^ mice (n=3 in each group).

**Figure S19.** The effect of *lncAPAT* on the mRNA expression of *CXCL9*, *CXCL10*, *CXCL11*, *CCL3,* and *CXCL4* in THP-1–derived macrophages (n=3 in each group).

**Table S1. Clinical characteristics of control subjects and coronary artery disease patients with mixed plaques on coronary computed tomography angiography.**

| **Characteristics** | **Control subjects**  **(n=10)** | **CAD patients**  **(n=5)** | ***P* value** |
| --- | --- | --- | --- |
| Age, years | 56.9 ± 6.4 | 59.8 ± 4.5 | 0.39 |
| Male, n (%) | 5 (50.0%) | 2 (40.0%) | 0.57 |
| Body mass index, kg/m^2^ | 24.4 ± 3.1 | 24.5 ± 3.6 | 0.96 |
| Laboratory data |  |  |  |
| Total cholesterol, mmol/L | 4.61 ± 0.93 | 5.07 ± 0.25 | 0.42 |
| Triglycerides, mmol/L | 1.22 (0.93-2.12) | 1.63 (0.99) | 0.61 |
| HDL-C, mmol/L | 1.29 ± 0.37 | 1.02 ± 0.22 | 0.27 |
| LDL-C, mmol/L | 2.67 ± 0.47 | 3.46 ± 0.13 | 0.02 |
| Plasma creatinine, µmol/L | 67.16 ± 9.30 | 72.22 ± 14.55 | 0.49 |

Data were given as mean ± SD, number (%), or median (interquartile range).

*P-value* was calculated by the chi-square test for categorical variables, the *t-test* for continuous variables, or the Mann-Whitney U test for triglycerides.

Abbreviations: CAD, coronary artery disease; HDL-C, high-density lipoprotein cholesterol; LDL-C, low-density lipoprotein cholesterol.

**Table S2. Clinical characteristics of control subjects, coronary artery disease patients with mixed plaques, and acute ST-elevation myocardial infarction patients undergoing optical coherence tomography examination.**

| **Characteristics** | **Control subject**  **(n= 37)** | **CAD patients**  **（n=22）** | **STEMI patients**  **(n= 22)** | ***P* value** |
| --- | --- | --- | --- | --- |
| Age, years | 51.7 ± 10.9 | 53.7 ± 3.2 | 58.1 ± 9.8 | 0.036 |
| Male, n (%) | 23 (62.2%) | 16（72.7%） | 19 (86.4) | 0.154 |
| Body mass index, kg/m^2^ | 25.6 ± 3.6 | 26.0 ± 2.4 | 26.2 ± 2.7 | 0.757 |
| Laboratory data |  |  |  | 0.433 |
| Total cholesterol, mmol/L | 4.6 ± 1.0 | 4.3 ± 0.9 | 4.3 ± 1.2 | 0.052 |
| Triglycerides, mmol/L | 1.7 (1.3, 2.5) | 1.5 (1.3, 1.8) | 1.3 (0.9, 1.6) | 0.018 |
| HDL-C, mmol/L | 1.3 ± 0.3 | 1.2 ± 0.3 | 1.0 ± 0.2 | 0.249 |
| LDL-C, mmol/L | 2.8 ± 0.8 | 2.5 ± 0.7 | 2.9 ± 0.8 | 0.210 |
| Plasma Creatinine, μmol/L | 76.7 ± 17.8 | 76.6 ± 13.8 | 77.5 ± 12.9 | 0.036 |

Data were given as mean ± SD, number (%), or median (interquartile range).

*P-value* was calculated by the chi-square test for categorical variables, the *t-test* for continuous variables, or the Mann-Whitney U test for triglycerides.

Abbreviations: CAD, coronary artery disease; STEMI, ST-elevation myocardial infarction; HDL-C, high-density lipoprotein cholesterol; LDL-C, low-density lipoprotein cholesterol.

**Table S3. The primers for** **quantitative real-time polymerase chain reaction analysis**

| **Species** | **Gene** | **Sequence (5’ −> 3’)** | |
| --- | --- | --- | --- |
| Human | *U2* | forward | CATCGCTTCTCGGCCTTTTG |
|  |  | reverse | CAGGTCCAGGGGTCTTGGTCC |
|  | *S14* | forward | GGCAGACCGAGATGAATCCTC |
|  |  | reverse | CAGGTCCAGGGGTCTTGGTCC |
|  | *lncAPAT* | forward | GACCACCGCCAACAGAAG |
|  |  | reverse | TGACAAAAGCCATAGCATGAA |
|  | *MCP-1* | forward | ATTCTTGGGTTGTGGAGTGAGTGTTCA |
|  |  | reverse | ATTCTTGGGTTGTGGAGTGAGTGTTCA |
|  | *ICAM1* | forward | TCTGTGTCCCCCCTCAAAAGTC |
|  |  | reverse | GGGTCTCTATGCCCAACAA |
|  | *IL-1β* | forward | CAGCTACGAATCTCCGACCAC |
|  |  | reverse | GGCAGGGAACCAGCATCTTC |
|  | *IL-10* | forward | AAGCCTTGTCTGAGATGAT |
|  |  | reverse | CATTCTTCACCTGCTCCA |
|  | *TNFα* | forward | CCGAGTGACAAGCCTGTA |
|  |  | reverse | GGACCTGGGAGTAGATGAG |
|  | *GAPDH* | forward | GAAGGTGAAGGTCGGAGTCA |
|  |  | reverse | GGAAGATGGTGATGGGATTTC |
|  | *RPL22* | forward | CACTCTTGATTGCACCCACCCT |
|  |  | reverse | CGGTGATCTTGCTCTTGCTCCT |
|  | *SR-A1* | forward | GATGCTCGCTCAATGACA |
|  |  | reverse | GCTGCCACTATTCCAATGA |
|  | *CD36* | forward | TGATGAACAGCAGCAACA |
|  |  | reverse | CACAGCCAGATTGAGAACT |
|  | *SR-B1* | forward | GCAACATCACCTTCAACAA |
|  |  | reverse | GGCTTATTCTCCATCATCAC |
|  | *ABCG1* | forward | CGGCTTCCTCTTCTTCTC |
|  |  | reverse | CCAGTAGTTCAGGTGTTCC |
| Mouse | *IL-6* | forward | AGAGGAGACTTCACAGAGG |
|  |  | reverse | GCATCATCGTTGTTCATA |
|  | *TNFα* | forward | CTCCCTCTCATCAGTTCTA |
|  |  | reverse | GTTGACTTTCTCCTGGTAT |
|  | *IL-1β* | forward | CTTCAGGCAGGCAGTATC |
|  |  | reverse | CAGCAGGTTATCATCATCATC |

**Continued.**

| **Species** | **Gene** | **Sequence (5’ −> 3’)** | |
| --- | --- | --- | --- |
| Mouse | *VCAM1* | forward | CCGAGCTAAATTACACATTGACCA |
|  |  | reverse | CATTGTCACAGCACCACCCTCT |
|  | *MMP9* | forward | GGATAAGGAGTTCTCTGGTGT |
|  |  | reverse | CACCTCATGGTCCACCTTGT |
|  | *COL1A1* | forward | CTGGAAGAGCGGAGAGTA |
|  |  | reverse | CTGTAGGTGAAGCGACTG |
|  | *COL3A1* | forward | CCTTCTACACCTGCTCCT |
|  |  | reverse | CCACTCCAGACTTGACATC |
|  | *GAPDH* | forward | TCTCCTGCGACTTCAACA |
|  |  | reverse | TGTAGCCGTATTCATTGTCA |

Abbreviations: *U2*: U2 small nuclear 1; *S14*: ribosomal protein S14; *LncAPAT*: atherosclerotic plaque instability associated transcript; *MCP-1*: monocyte chemoattractant protein 1; *ICAM1*: intercellular adhesion molecule 1; *IL-1β*: interleukin 1 β; *IL-10*: interleukin 10; *TNFα*: tumor necrosis factor α; *GAPDH*: glyceraldehyde-3-phosphate dehydrogenase; *RPL22*: ribosomal protein L22; *SR-A1*: scavenger receptor A1; *CD36*: CD36 molecule; *SR-B1*: scavenger receptor B1; *ABCG1*: ATP binding cassette subfamily G member 1; *IL-6*: interleukin 6; *VCAM1*: vascular cell adhesion molecule 1; *MMP9*: matrix metalloproteinase 9; *COL1A1*: collagen type I alpha 1 chain; *COL3A1*: collagen type III alpha 1 chain.

**Table S4.** **Potential targets of *lncAPAT* enriched by chromatin isolation by RNA purification assay (ChIRP).**

| **Gene ID** | **Symbol** | **Description** | **Fold enrichment** | | **Gene position** |
| --- | --- | --- | --- | --- | --- |
| 6146 | *RPL22* | ribosomal protein L22 | | 4.93263 | promoter |
| 64089 | *SNX16* | sorting nexin 16 | | 12.6373 | promoter |
| 158067 | *AK8* | adenylate kinase 8 | | 8.31048 | promoter |
| 5725 | *PTBP1* | polypyrimidine tract binding protein 1 | | 4.92964 | promoter |
| 388591 | *RNF207* | ring finger protein 207 | | 4.93263 | promoter |


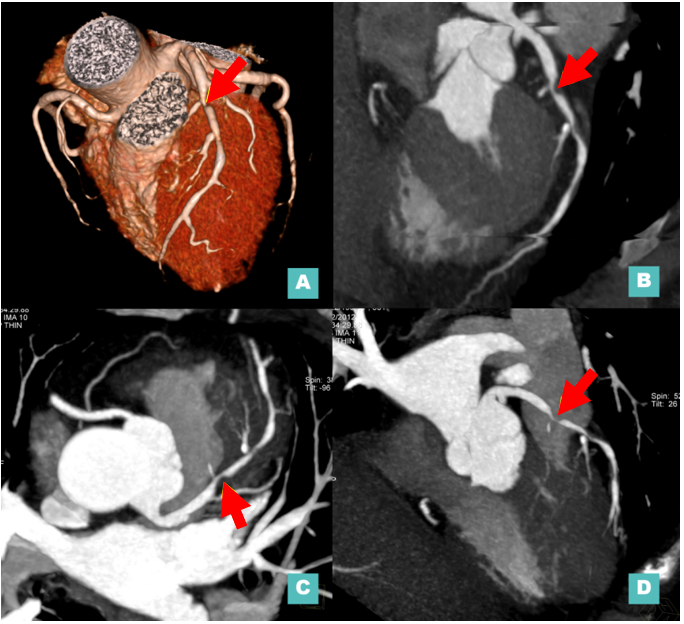


**Figure S1. The representative image of coronary artery disease patients with mixed plaques on coronary computed tomography angiography.**

**A.** VR (anterior wall of the heart) showed eccentric stenosis of >70% in the middle segment of the anterior descending artery. **B.** Curved surface reconstruction showed a biased noncalcified plaque in the middle of the anterior descending branch (red arrow). **C.** MIP view of the anterior wall of the heart. **D.** septal view shows eccentric stenosis >70% in the middle of the anterior descending branch (red arrow).


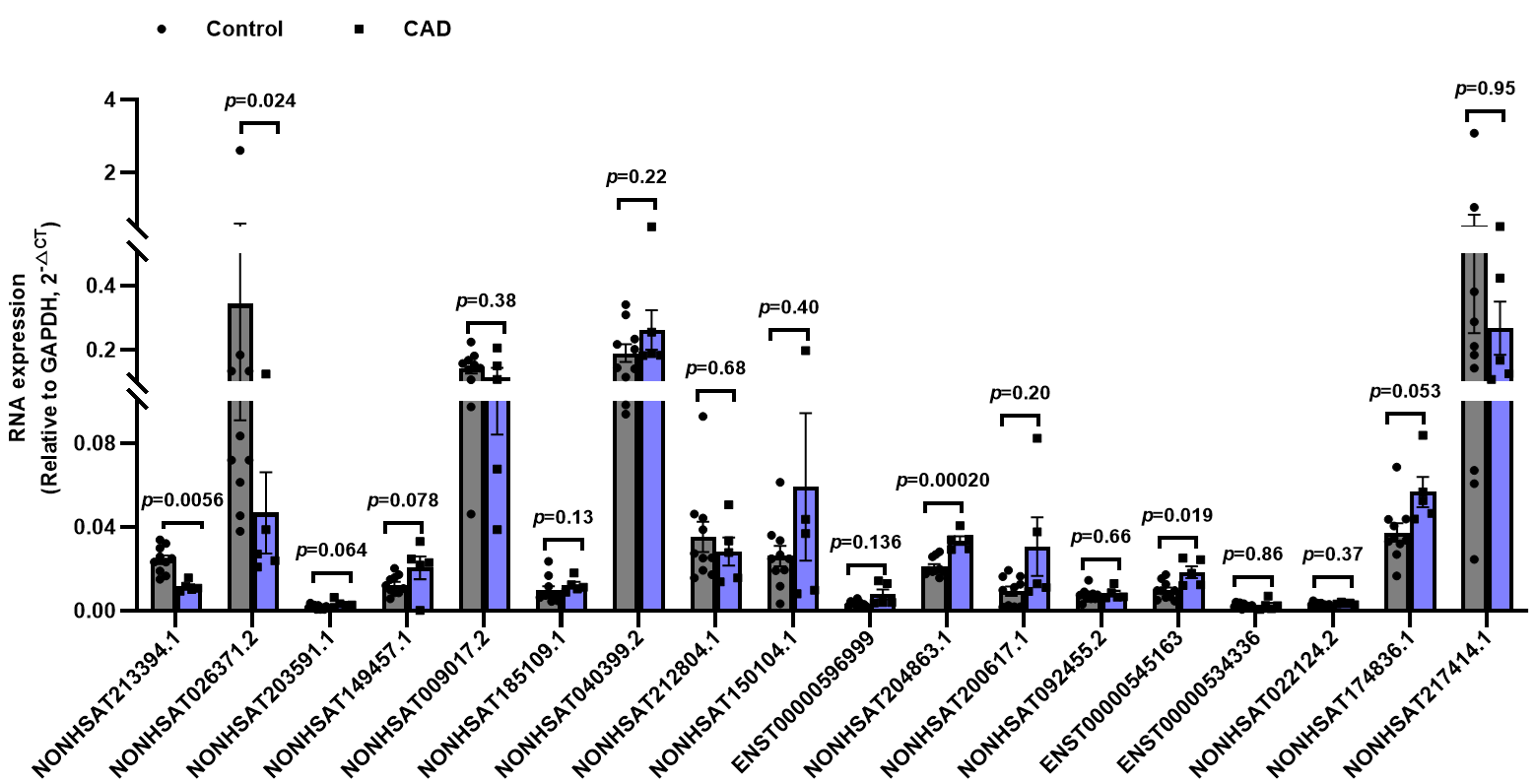


**Figure S2. The validation of candidate transcripts by the qRT-PCR method in the peripheral blood of coronary artery disease (CAD) patients (n=5) who had coronary mixed plaques with coronary computer tomography angiography, compared with control subjects (n=10).** Grey bars represent control subjects, blue bars represent CAD patients. Data are shown as mean ± SEM. Statistical analysis was conducted using the Mann-Whitney U test.


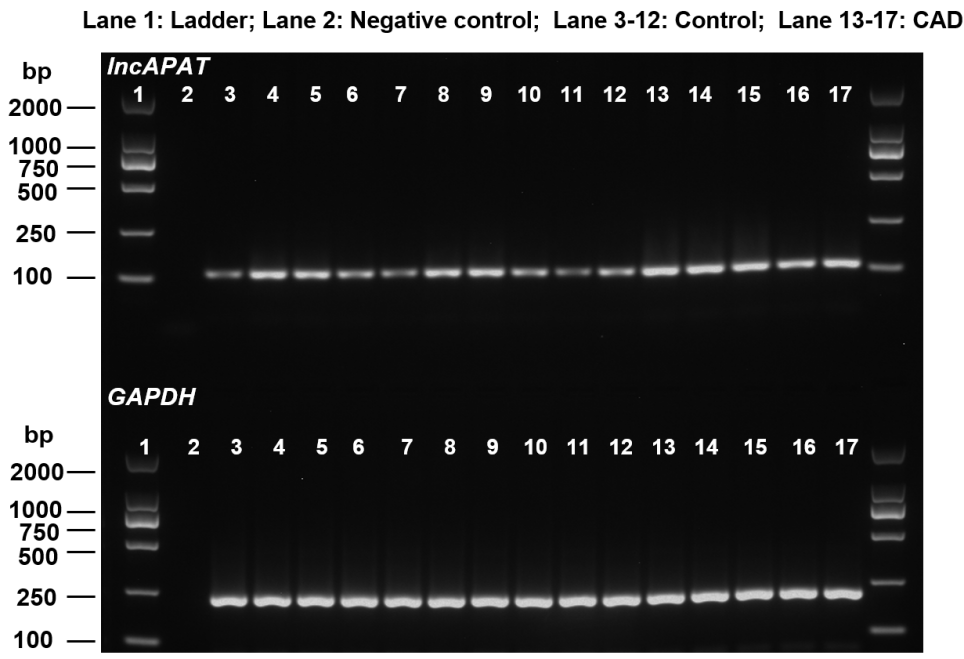


**Figure S3. Agarose gel electrophoresis of RT-PCR products for *lncAPAT* and *GAPDH* in the peripheral blood of coronary artery disease patients (n=5) who had coronary mixed plaques with coronary computer tomography angiography and control subjects (n=10).**


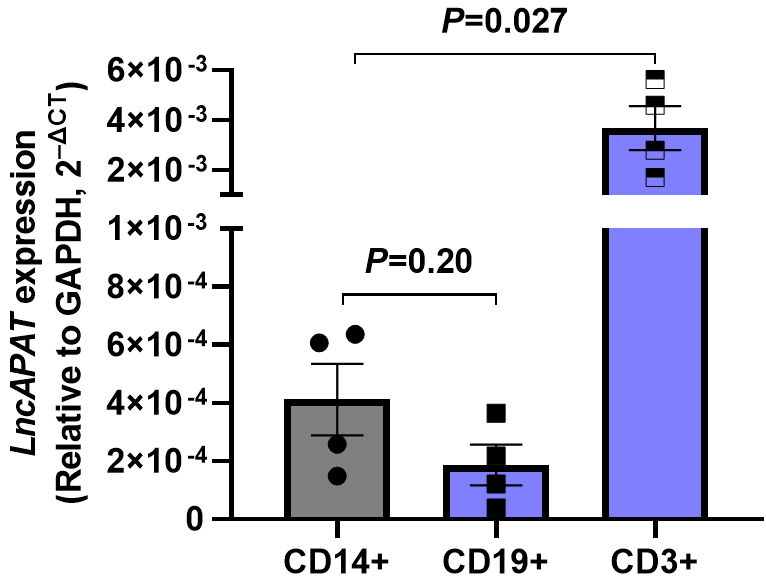


**Figure S4. The expression of *lncAPAT* in monocytes (CD14+), B cells (CD19+), and T cells (CD3+) isolated by magnetic bead sorting from the peripheral blood of control subjects (n=4).**

Data are shown as mean ± SEM. Statistical analysis was conducted using one-way ANOVA with Tukey’s multiple comparisons test.


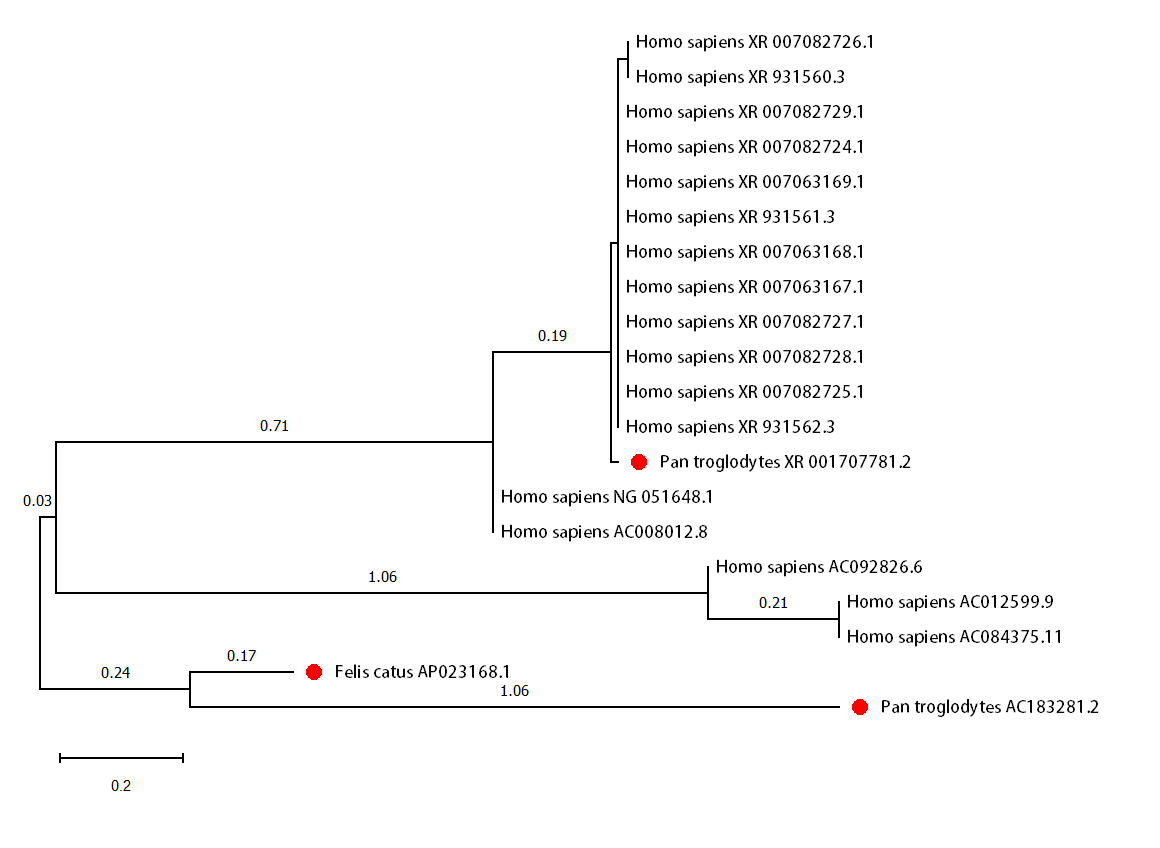


**Figure S5. The phylogenetic tree of *lncAPAT*.**


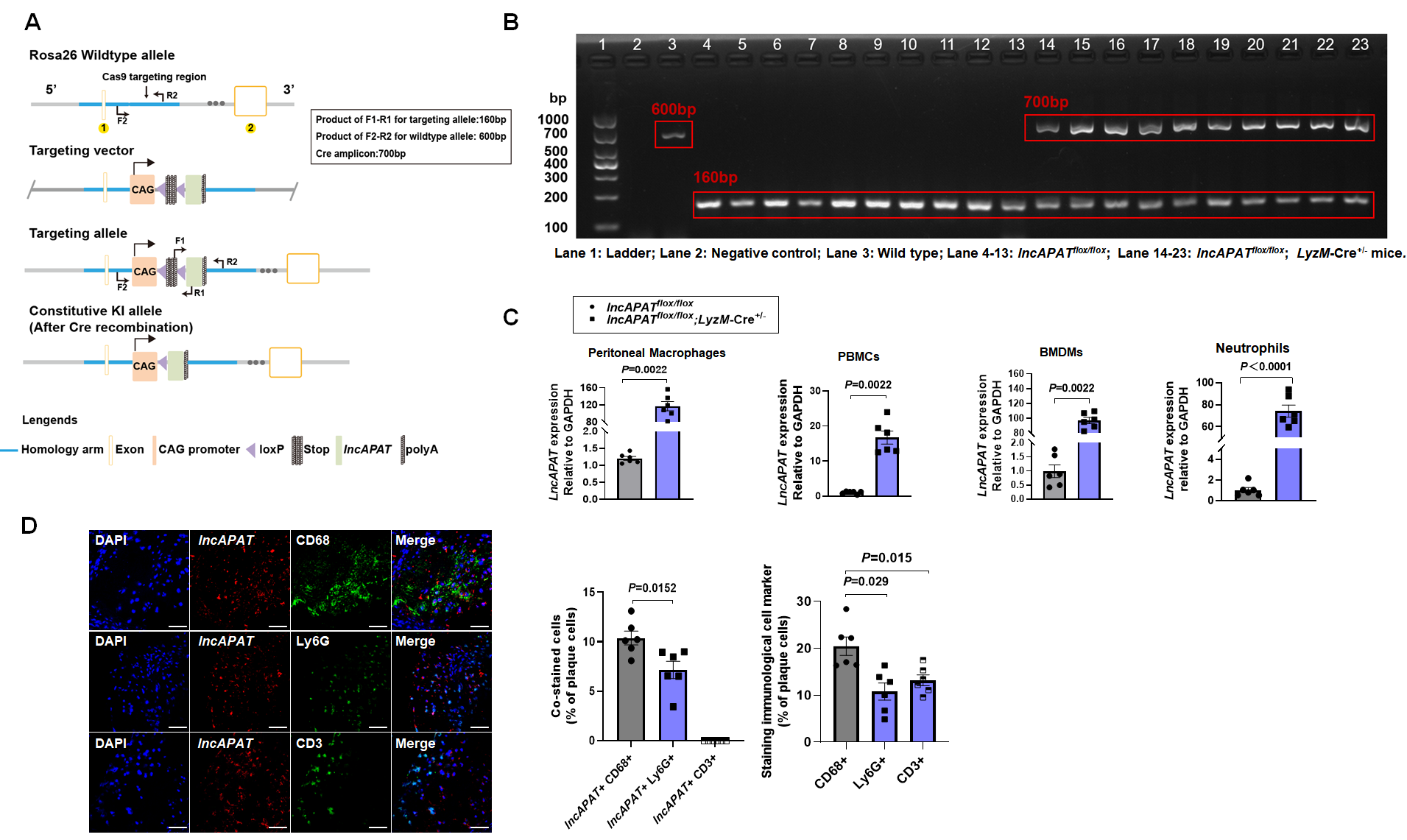


**Figure S6. Establishment and validation of myeloid cell-specific *lncAPAT* knock-in mice.**

**A,** The schematic overview of the *lncAPAT* overexpression strategy in mice. **B,** The genotype of *lncAPAT^flox/flox^* mice and *lncAPAT^flox/flox^;LyzM*-Cre^+/-^ mice using genomic DNA extracted from mouse tails (n=10 in each group). **C,** The efficiency of *lncAPAT* overexpression in peritoneal macrophages, peripheral blood mononuclear cells (PBMCs), bone marrow-derived macrophages (BMDMs), and neutrophils (n=6 in each group). **D,** Representative images of fluorescence in situ hybridization for *lncAPAT* (red) and immunostaining for CD68 (green), Ly6G (green), CD3 (green), along with 4′,6-diamidino-2-phenylindole (DAPI) nuclear stain (blue) in thoracic aorta plaques of *lncAPAT^flox/flox^;LyzM*-Cre^+/-^ mice (n=6 in each group). Scale bar=50 µm.

Data are shown as mean ± SEM. Mann-Whitney U test was used for C, and one-way ANOVA with Tukey’s multiple comparisons test was used for D.


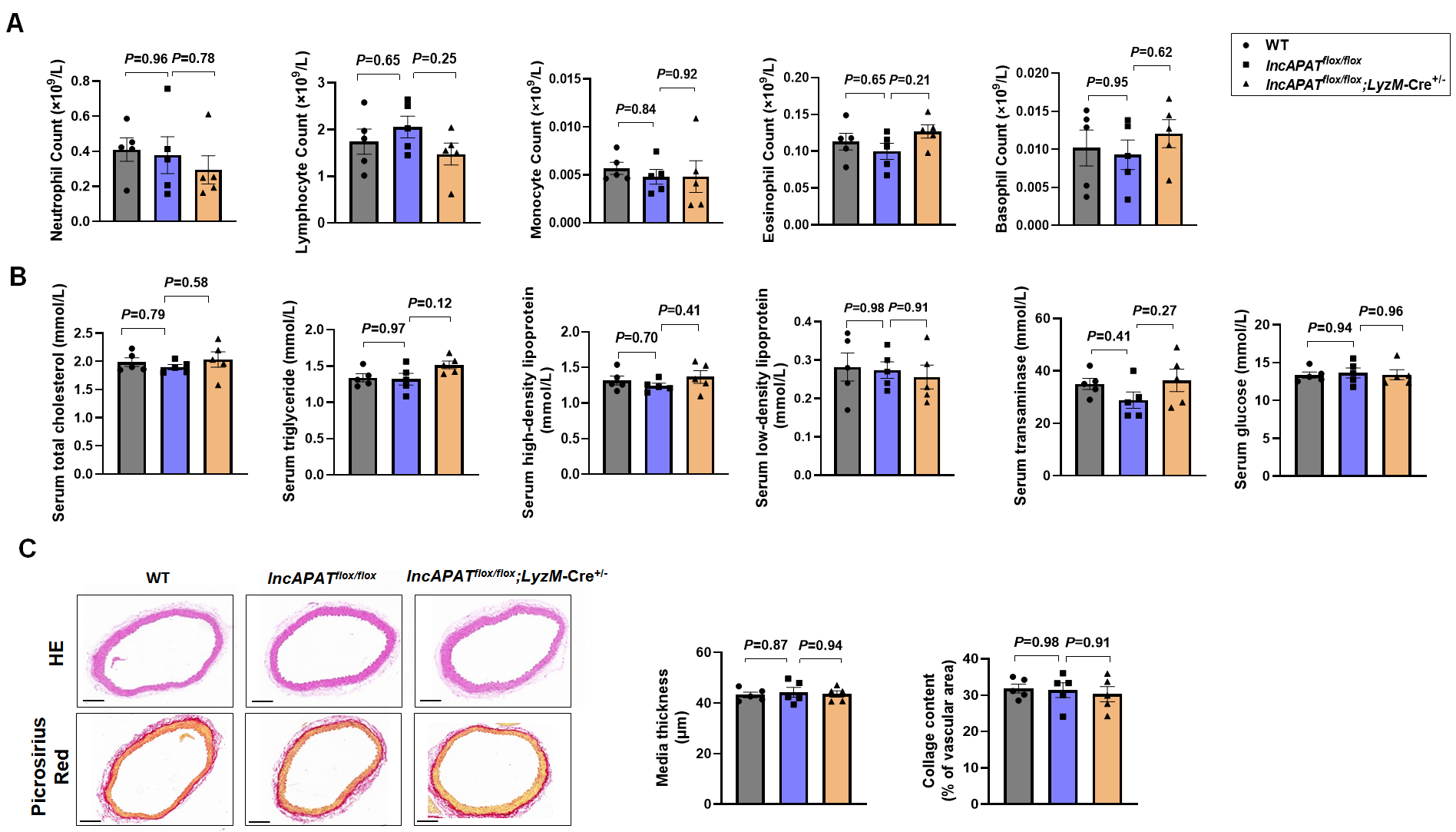


**Figure S7. The effect of *lncAPAT* on baseline phenotypes of wild-type, *lncAPAT^flox/flox,^* and *lncAPAT^flox/flox^;LyzM*-Cre^+/-^ mice before experimental intervention.**

**A,** The immune cell composition analysis of neutrophils, lymphocytes, monocytes, eosinophils, and basophils (n=5 in each group). **B,** The serum biochemical analysis of total cholesterol**,** triglyceride**,** high-density lipoprotein**,** low-density lipoprotein**,** transaminase, and glucose (n=5 in each group). **C,** Representative images of Hematoxylin-Eosin and Sirius Red staining, and quantification of media thickness and collagen content in the cross sections of the thoracic aorta (n=5 in each group). Scale bar=100 µm.

Gray bars represent wild-type (WT) mice, blue bars represent *lncAPAT^flox/flox^* mice, and orange bars represent *lncAPAT^flox/flox^;LyzM*-Cre^+/^**^-^** mice. Data are shown as mean ± SEM. Statistical analysis was conducted using one-way ANOVA with Tukey’s multiple comparisons test.


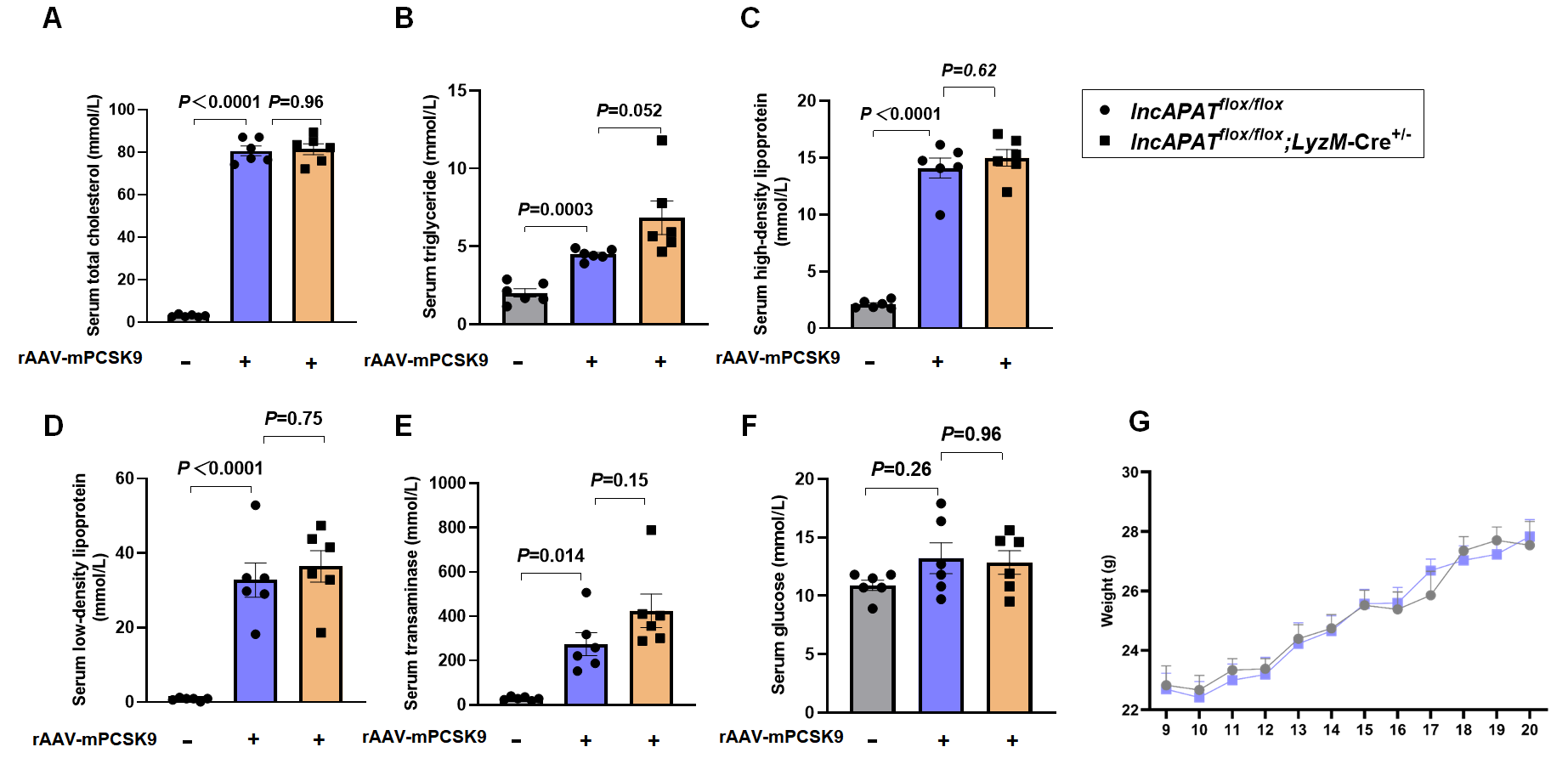


**Figure S8. The effect of *lncAPAT* on serum biochemical markers and body weight of *lncAPAT^flox/flox^* mice and *lncAPAT^flox/flox^;LyzM*-Cre^+/-^ mice.**

**A-F,** The serum biochemical analysis of total cholesterol **(A),** triglyceride **(B),** high-density lipoprotein **(C),** low-density lipoprotein **(D),** transaminase, and glucose **(F)** (n=6 in each group)**.** **G**, Body weight of mice during western diet feeding for 12 weeks (n=20 in each group). Data are shown as mean ± SEM.

Gray bars represent *lncAPAT^flox/flox^* mice not injected with rAAV-mPCSK9, blue and orange bars represent *lncAPAT^flox/flox^* and *lncAPAT^flox/flox^;LyzM*-Cre^+/-^ mice injected with rAAV-mPCSK9, respectively. Data are shown as mean ± SEM. Statistical analysis was conducted using one-way ANOVA with Tukey’s multiple comparisons test.


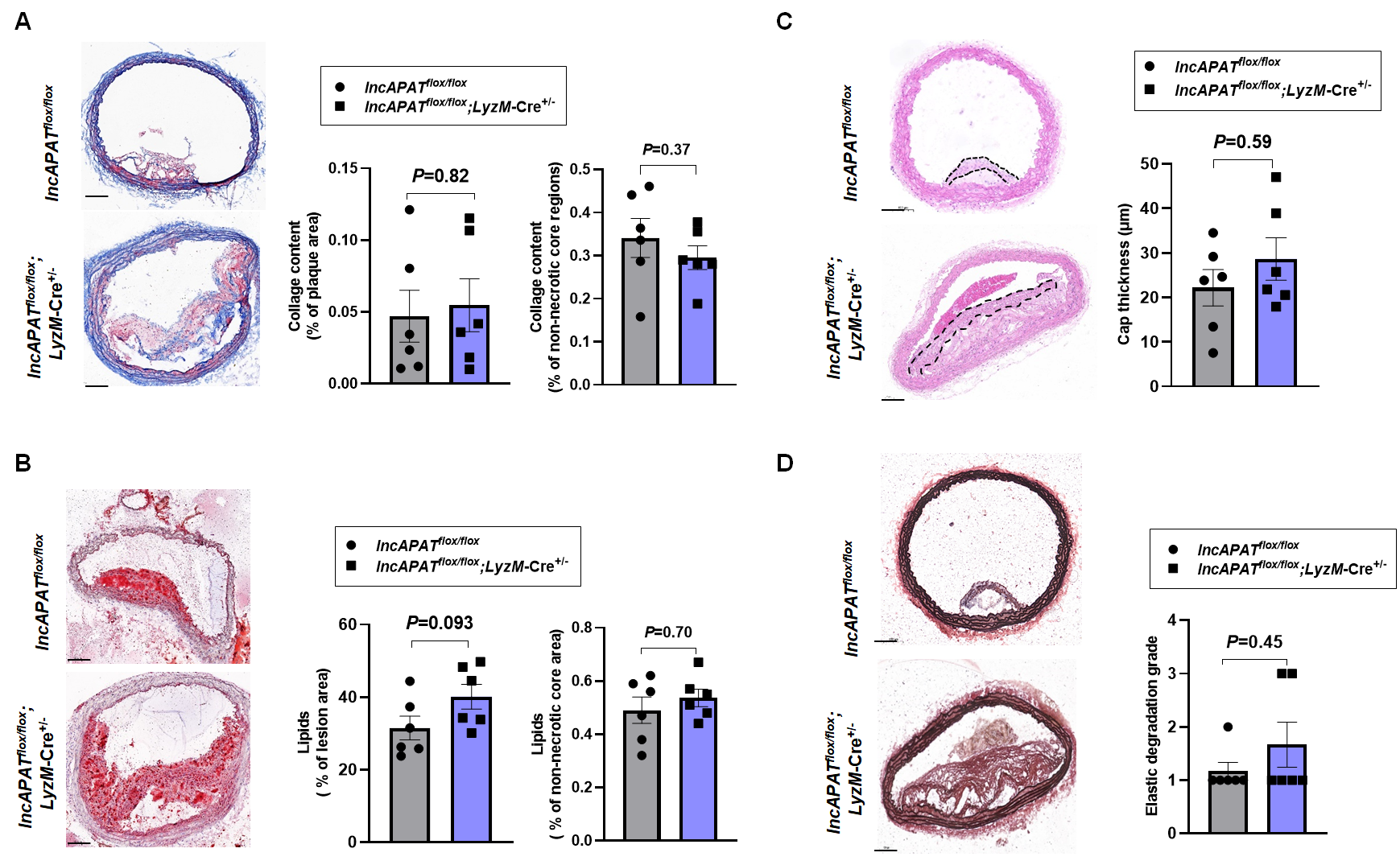


**Figure S9. The effect of *lncAPAT* on collagen content, lipid deposition, cap thickness, and elastic degradation grade in the thoracic aorta of the *lncAPAT^flox/flox^* mice and *lncAPAT^flox/flox^;LyzM*-Cre^+/-^ mice.**

**A,** Representative images of Masson’s trichrome and quantification of collagen content in the cross sections of thoracic aorta (n=6 in each group). Scale bar=100 μm. **B,** Representative images of Oil-Red O staining and quantification of lipid content in the cross sections of thoracic aorta (n=6 in each group). Scale bar=100 μm. **C,** Representative images of hematoxylin-eosin and quantification of cap thickness in the cross sections of thoracic aorta (n=6 in each group). Scale bar=100 μm. **D,** Representative images and quantification of elastic degradation in the cross sections of thoracic aorta (n=6 in each group). Scale bar=100 μm.

Grey bars represent *lncAPAT^flox/flox^* mice, blue bars represent *lncAPAT^flox/flox^;LyzM*-Cre^+/-^ mice. Data are shown as mean ± SEM. Statistical analysis was conducted using Mann-Whitney U test.


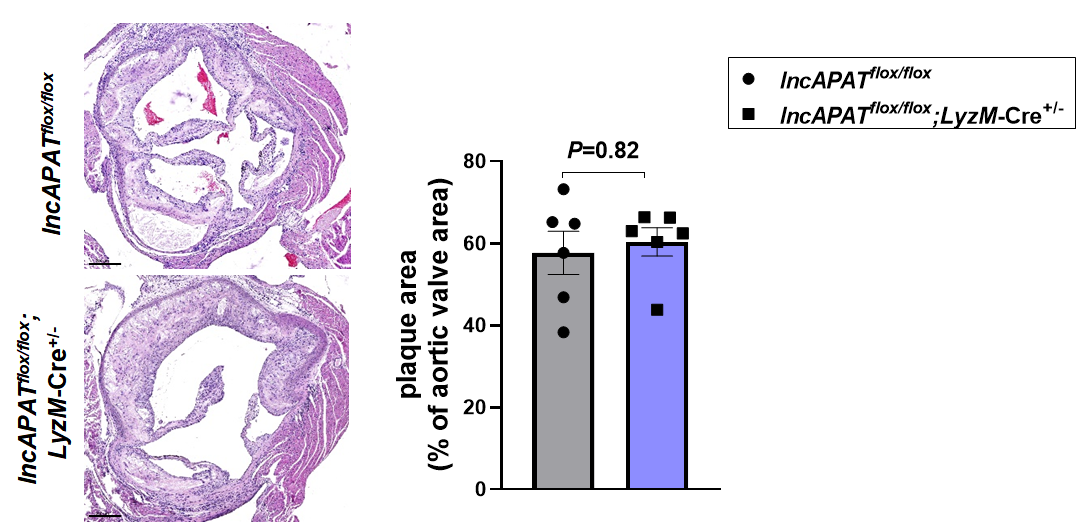


**Figure S10. The effect of *lncAPAT* on atherosclerotic lesion areas of the aorta root of the *lncAPAT^flox/flox^* mice and *lncAPAT^flox/flox^;LyzM*-Cre^+/-^ mice.**

Representative images of hematoxylin-eosin staining for morphological analysis of plaque lesion areas (n=6 in each group). Scale bar = 200 μm. Grey bars represent *lncAPAT^flox/flox^* mice, blue bars represent *lncAPAT^flox/flox^;LyzM*-Cre^+/-^ mice. Data are shown as mean ± SEM. Statistical analysis was conducted using Mann-Whitney U test.


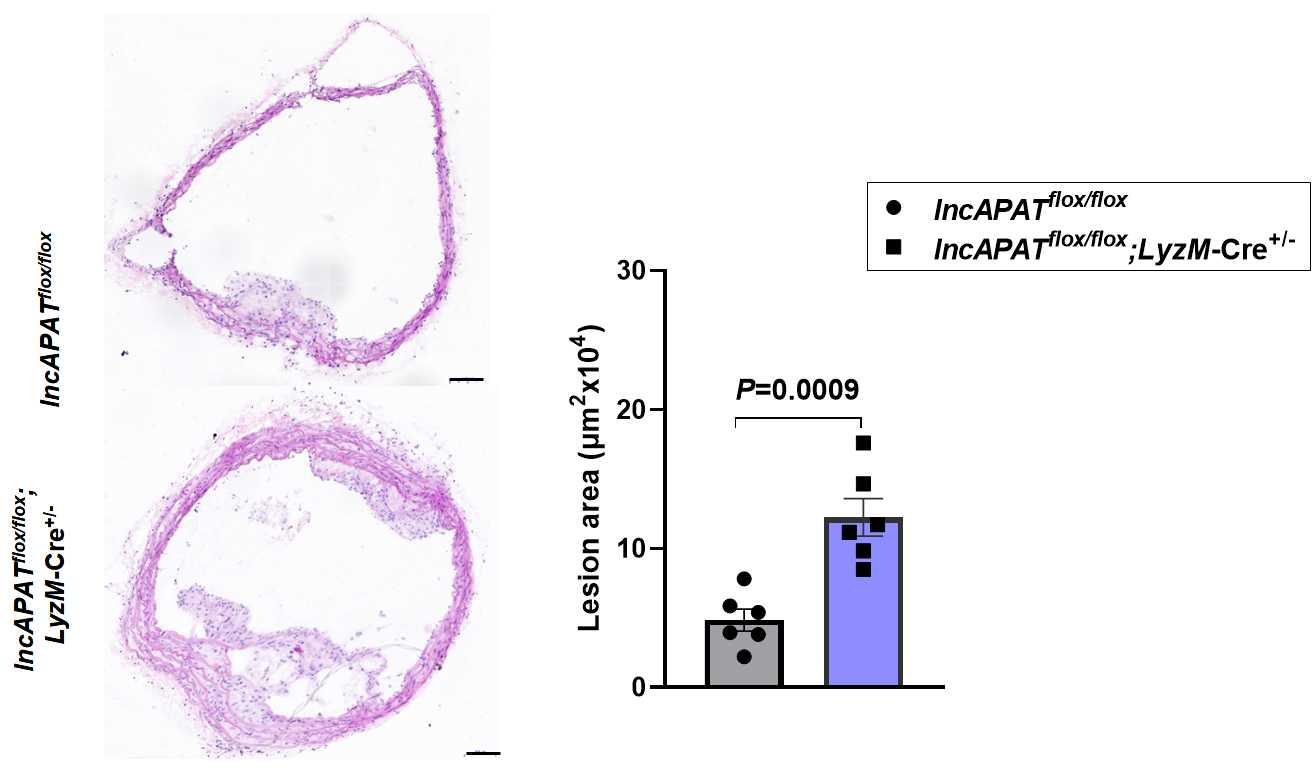


**Figure S11. The effect of *lncAPAT* on atherosclerotic lesion areas of** **the thoracic aorta in female *lncAPAT^flox/flox^* mice and *lncAPAT^flox/flox^;LyzM*-Cre^+/-^ mice.**

Representative images of hematoxylin-eosin staining for morphological analysis of plaque lesion areas in female *lncAPAT^flox/flox^* mice and *lncAPAT^flox/flox^;LyzM*-Cre^+/-^ mice (n=6 in each group). Scale bar=200 μm. Grey bars represent *lncAPAT^flox/flox^* mice, blue bars represent *lncAPAT^flox/flox^;LyzM*-Cre^+/-^ mice. Data are shown as mean ± SEM. Statistical analysis was conducted using the 2-tailed unpaired t-test.


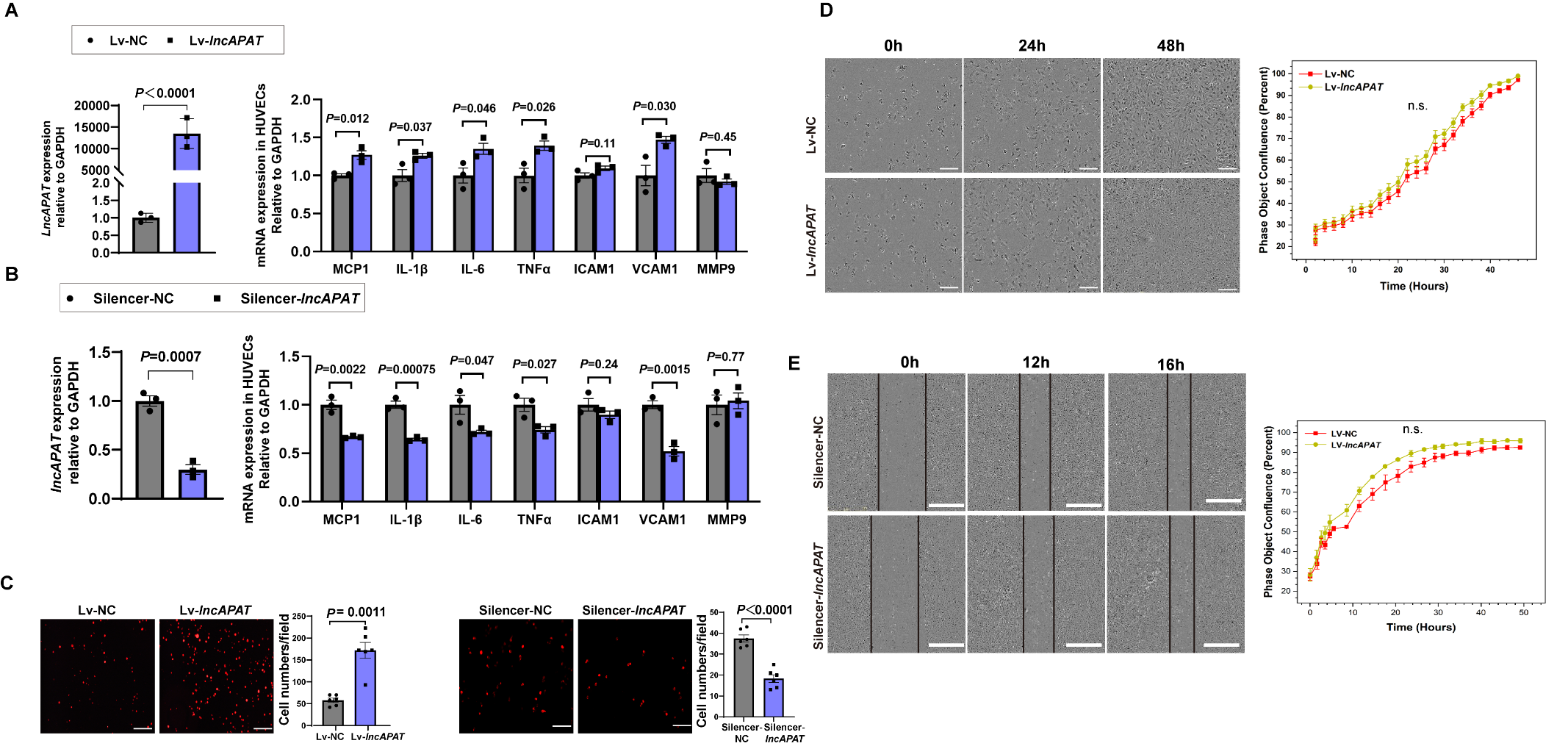


**Figure S12. Effects of *lncAPAT* on the function of human umbilical vein endothelial cells.**

**A-B,** The mRNA expression of *MCP-1, IL-1β, IL-6, TNFα, ICAM1, VCAM1,* and *MMP9* in human umbilical vein endothelial cells (HUVECs) with overexpression of *lncAPAT* by lentivirus (n=3 in each group) **(A)** or knockdown of *lncAPAT* by smart silencer (n=3 in each group) **(B)**. **C,** Representative images and quantification of THP-1 monocytes adhering to human umbilical vein endothelial cells (HUVECs) with overexpression of *lncAPAT* by lentivirus (n=6 in each group) or knockdown of *lncAPAT* by smart silencer (n=6 in each group). Scale bar=200 μm. **D,** Representative images and quantification of HUVECs at 0, 24, and 48 h with overexpression of *lncAPAT* by lentivirus in proliferation assay (n=3 in each group). Scale bar=200 μm. **E,** Representative images and quantification of HUVECs at 0, 12, and 16 h with overexpression of *lncAPAT* by lentivirus in migration assay (n=3 in each group). Scale bar=200 μm.

Grey bars represent controls, blue bars represent experiments. Data are mean ± SEM. The 2-tailed unpaired t-test was used for A, B, D, and E. The Mann-Whitney U test was used for C.


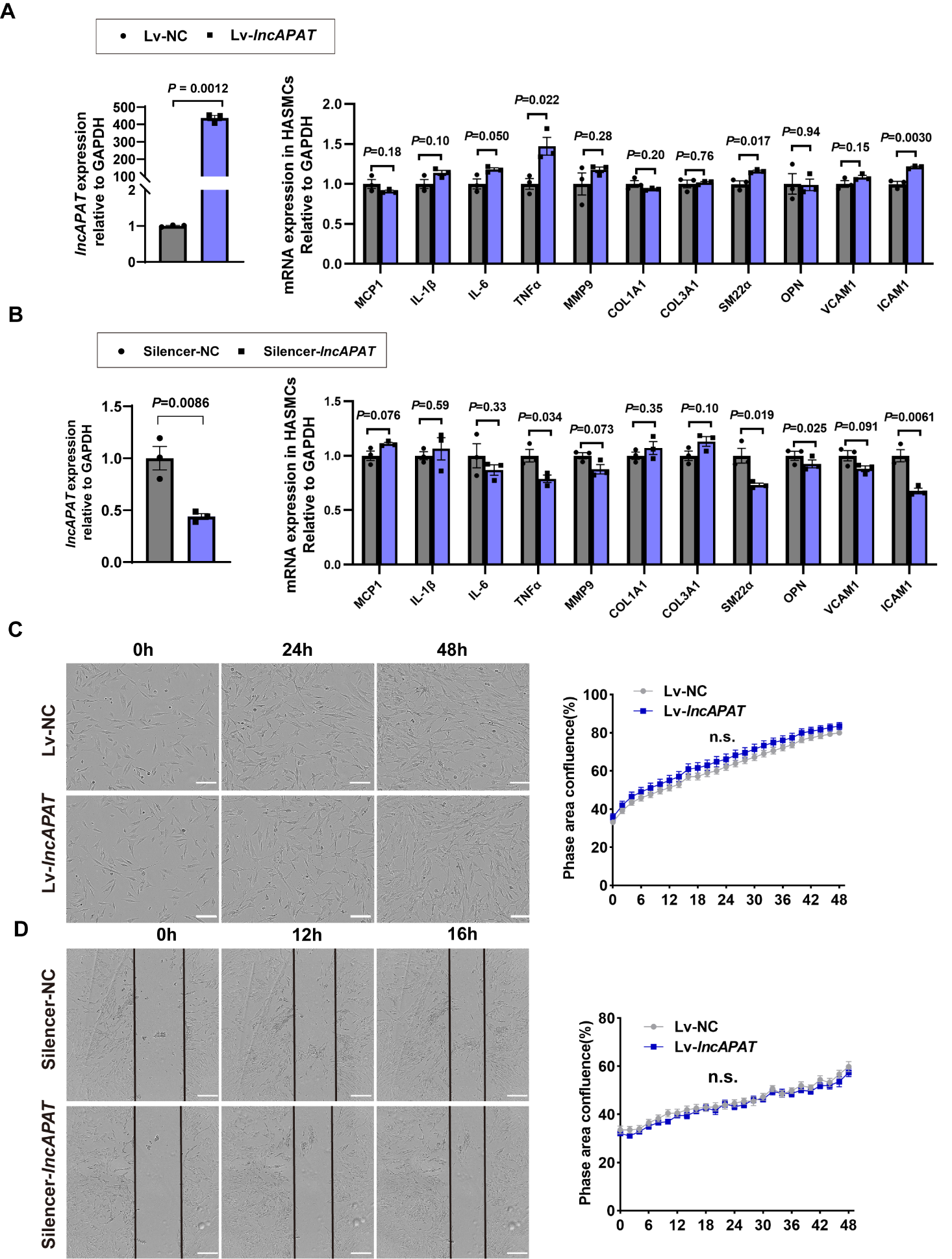


**Figure S13. Effects of *lncAPAT* on the function of human aortic smooth muscle cells.**

**A-B,** The mRNA expression of *MCP-1, IL-1β, IL-6, TNFα, MMP9, COL1A1, COL3A1, SM22α, OPN, VCAM1,* and *ICAM1* in human aortic smooth muscle cells (HASMCs) with overexpression of *lncAPAT* by lentivirus (n=3 in each group) **(A)** or knockdown of *lncAPAT* by smart silencer (n=3 in each group) **(B)**. **C,** Representative images and quantification of HASMCs at 0, 24, and 48 h with overexpression of *lncAPAT* by lentivirus in the proliferation assay (n=3 in each group). Scale bar=200 μm. **D,** Representative images and quantification of HUVECs at 0, 12, and 16 h with overexpression of *lncAPAT* by lentivirus in migration assay (n=3 in each group). Scale bar=200 μm.

Grey bars represent controls, blue bars represent experiments. Data are mean ± SEM. Statistical analysis was conducted using the 2-tailed unpaired t-test.


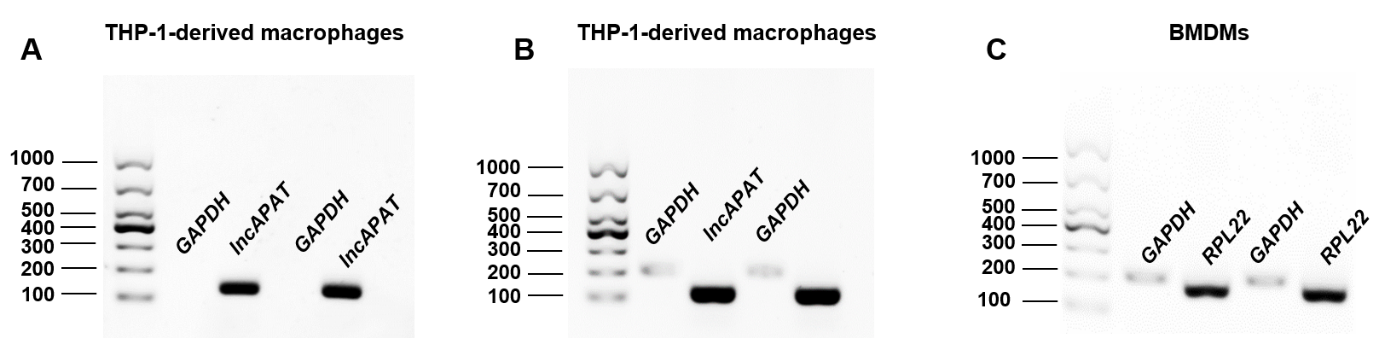


**Figure S14. Agarose gel electrophoresis of RT-PCR products from the ChIRP assay.**

**A-B,** Agarose gel electrophoresis of RT-PCR products for *GAPDH* and *lncAPAT* from the ChIRP assay in THP-1-derived macrophages (n=2 in each group) related to Figure 6A **(A)**, and bone marrow-derived macrophages (BMDMs) of *lncAPAT^flox/flox^;LyzM*-Cre^+/-^ mice (n=2 in each group) related to Figure 6D **(B)**. **C,** Agarose gel electrophoresis of RT-PCR products for *GAPDH* and *RPL22* from BMDMs of *lncAPAT^flox/flox^;LyzM*-Cre^+/-^ mice (n=2 in each group) related to Figure 6D.


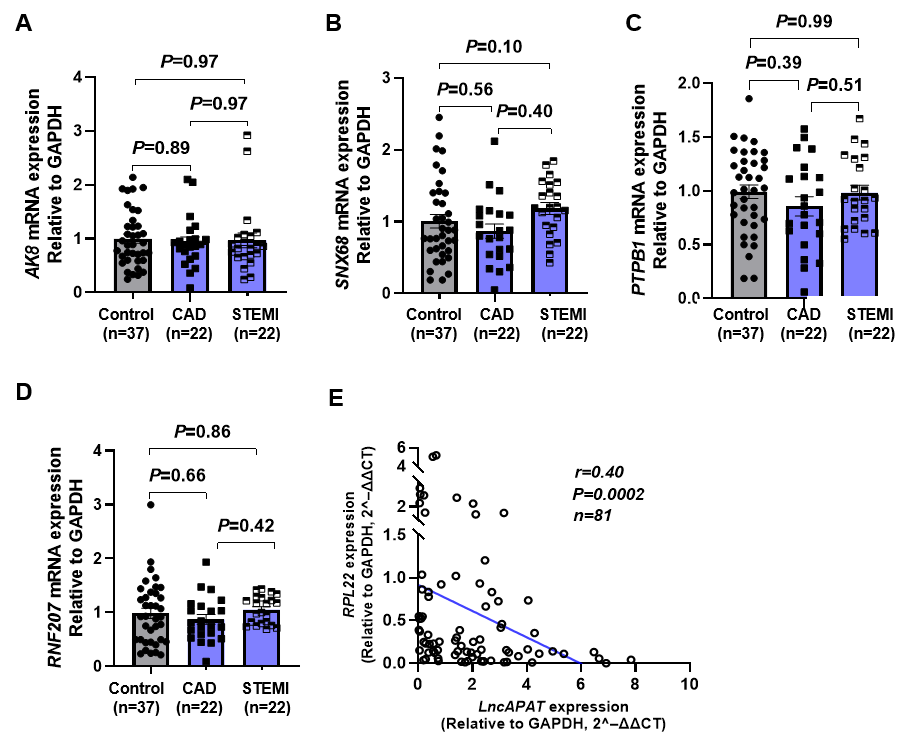


**Figure S15. The mRNA expression of potential targets of *lncAPAT* in the peripheral blood of**

**coronary artery disease (CAD) patients and acute ST-elevation myocardial infarction (STEMI) patients.**

**A– D,** qRT-PCR analysis of *AK8, SNX8, PTPB1,* and *RNF207* mRNA levels in peripheral blood of CAD patients with coronary computed tomography angiography examination (n=22), acute ST-elevation myocardial infarction (STEMI) patients with optical coherence tomography examination (n=22), and control subjects (n=37). **E,** Correlation between *lncAPAT* and *RPL22* mRNA expression in the peripheral blood of these participants (n = 81).

Grey bars represent control subjects, blue bars represent CAD patients, and orange bars represent STEMI patients. Data are shown as mean ± SEM. Kruskal-Wallis test followed by Dunn’s multiple comparison tests was used for A to D. Spearman correlation analysis was used for E.


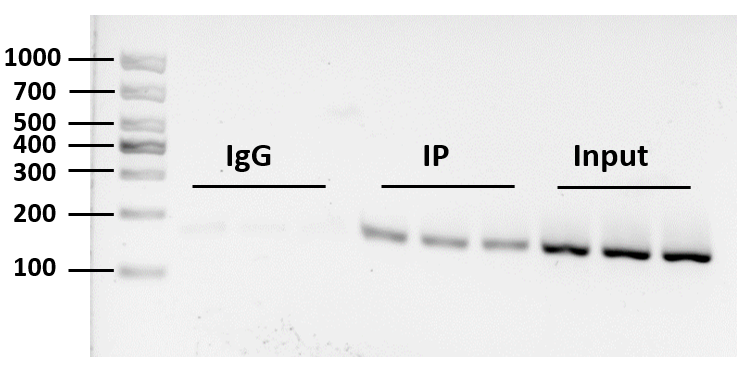


**Figure S16. Agarose gel electrophoresis of RT-PCR products for *MCP-1* from the RIP assay in bone marrow-derived macrophages of *lncAPAT^flox/flox^;LyzM*-Cre^+/-^ mice (n=3 in each group).**


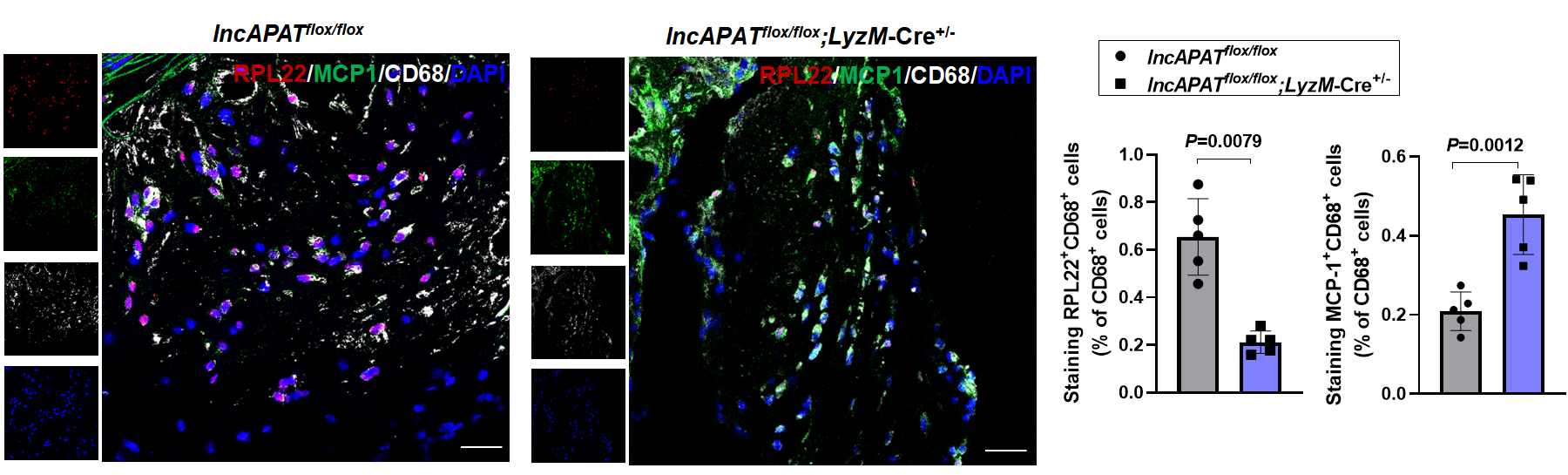


**Figure S17. The effect of *lncAPAT* on the expression of RPL22 and MCP-1 in macrophages of thoracic aorta plaque in female *lncAPAT^flox/flox^* mice and *lncAPAT^flox/flox^;LyzM*-Cre^+/-^ mice.**

Representative images and quantification of immunostaining for RPL22 (red), MCP-1 (green), CD68 molecule (white), and 4′,6-diamidino-2-phenylindole (DAPI) nuclear stain (blue) in thoracic aorta plaques of female *lncAPAT^flox/flox^* and *lncAPAT^flox/flox^;LyzM*-Cre^+/^**^-^** mice (n=5 in each group). Scale bar = 25 µm. Grey bars represent *lncAPAT^flox/flox^* mice, blue bars represent *lncAPAT^flox/flox^;LyzM*-Cre^+/^**^-^** mice. Data are shown as mean ± SEM. Statistical analysis was conducted using the 2-tailed unpaired t-test.


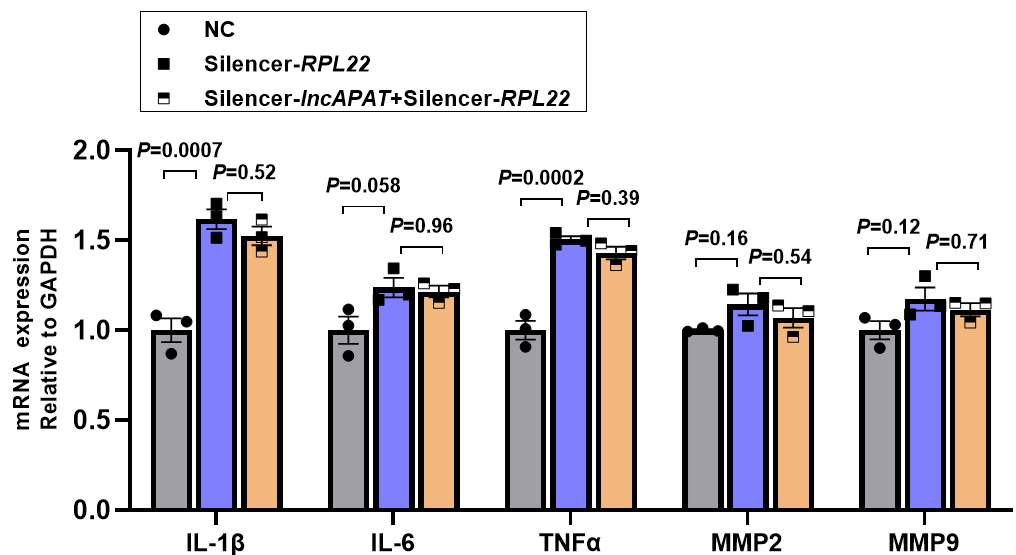


**Figure S18. The effect of *lncAPAT* –*RPL22* axis on the mRNA expression of *IL-6*, *IL-1β*, *TNFα*, *MMP-2*, and *MMP-9* in bone marrow-derived macrophages of *lncAPAT^flox/flox^;LyzM*-Cre^+/-^ mice (n=3 in each group).**

Grey bars represent the control group, blue or orange bars represent the experimental group. Statistical analysis was conducted using One-way ANOVA with Tukey’s multiple comparisons test.


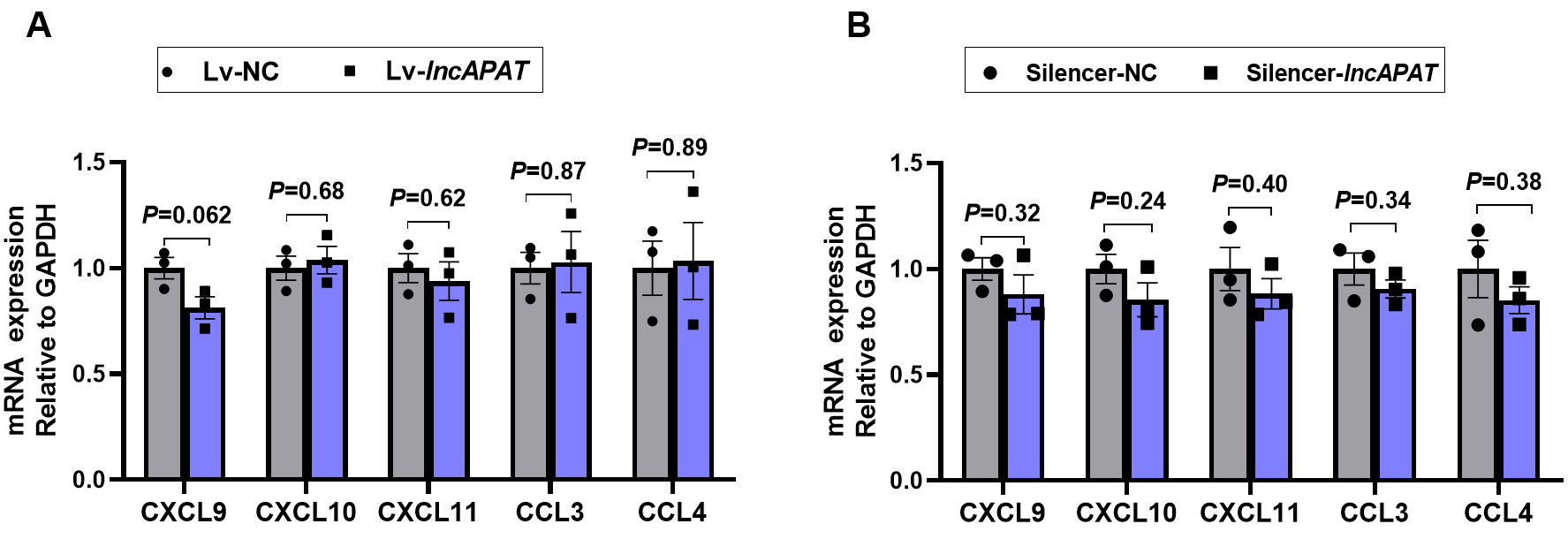


**Figure S19. The effect of *lncAPAT* on the mRNA expression of *CXCL9*, *CXCL10*, *CXCL11*, *CCL3,* and *CXCL4* in THP-1–derived macrophages (n=3 in each group).**

Grey bars represent the control group, blue bars represent the experimental group. Statistical analysis was conducted using a 2-tailed unpaired Student t-test.

**WB raw data**

The black box represents the graph used in the statistical analysis. The red box represents the graph presented within the manuscript.


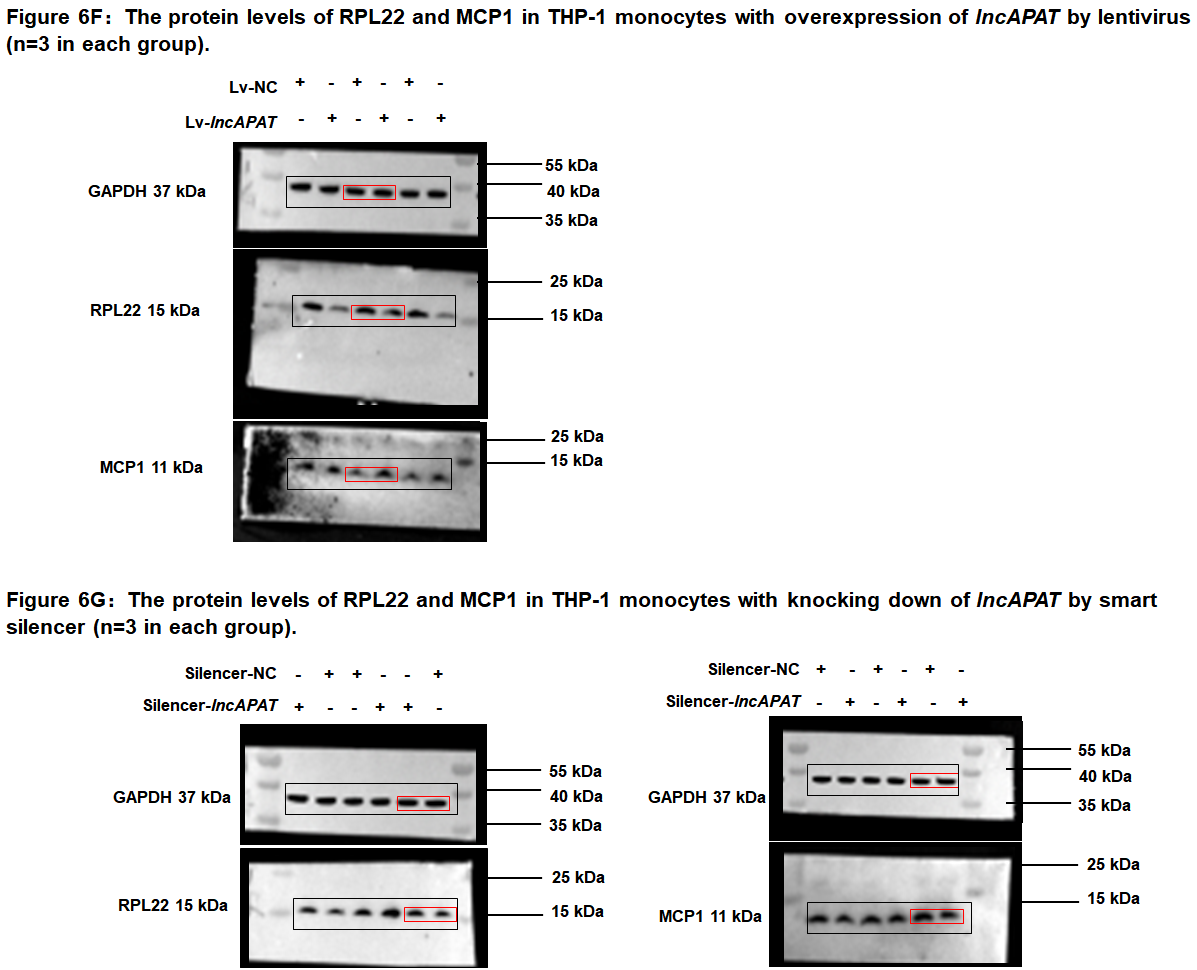


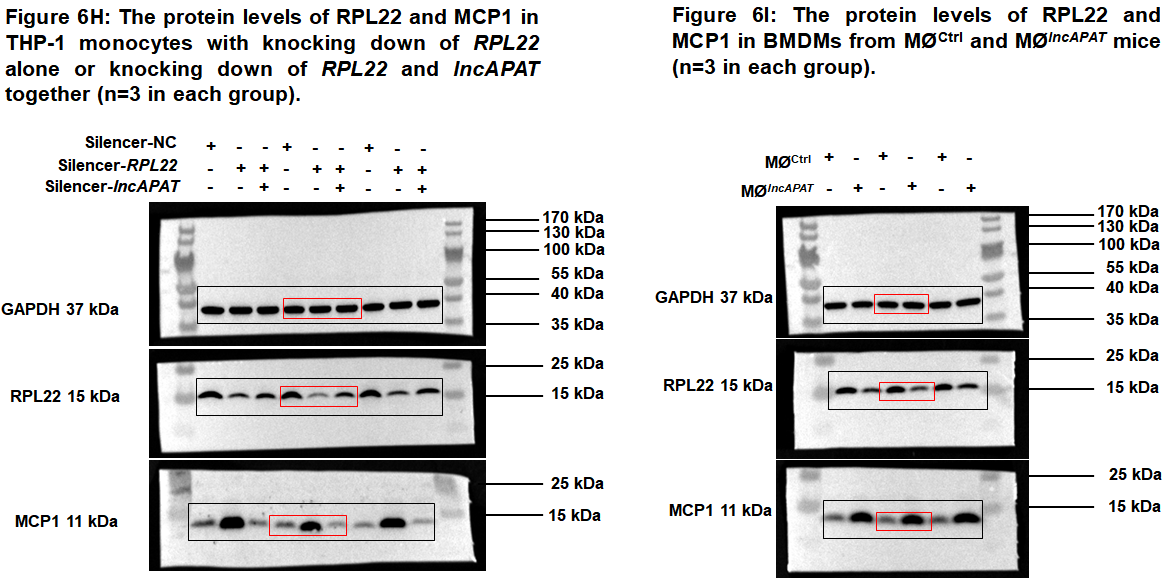


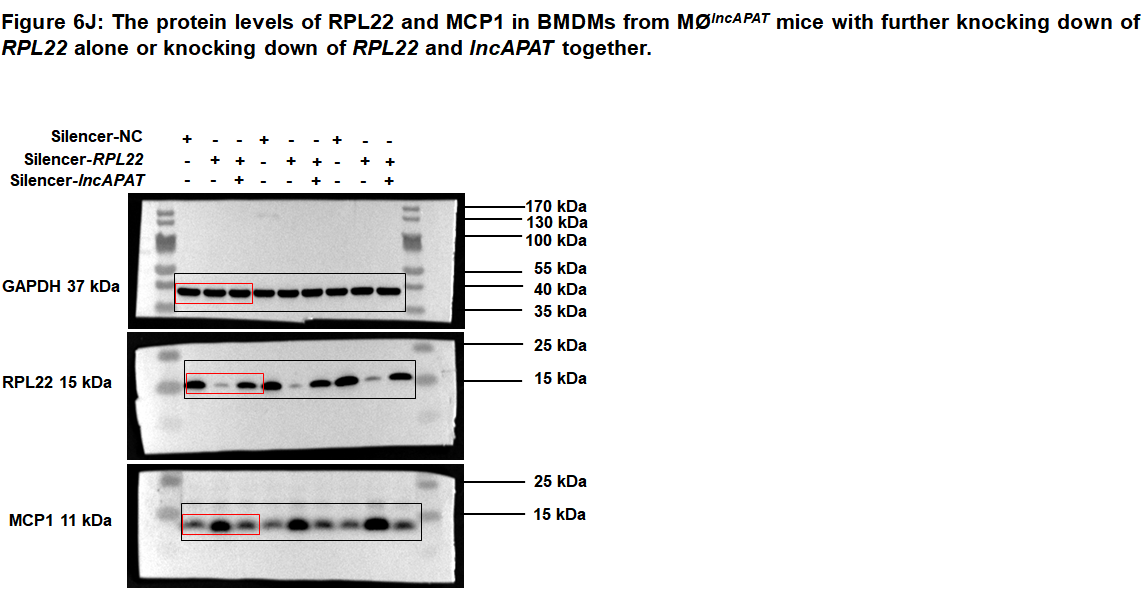

Supplement: Supplementary file 1 — Supporting Information [file CTM2-16-e70564-s001.docx]
